# Molecular hybridization strategy for tuning bioactive peptide function

**DOI:** 10.1038/s42003-023-05254-7

**Published:** 2023-10-19

**Authors:** Cibele Nicolaski Pedron, Marcelo Der Torossian Torres, Cyntia Silva Oliveira, Adriana Farias Silva, Gislaine Patricia Andrade, Yiming Wang, Maria Aparecida Silva Pinhal, Giselle Cerchiaro, Pedro Ismael da Silva Junior, Fernanda Dias da Silva, Ravi Radhakrishnan, Cesar de la Fuente-Nunez, Vani Xavier Oliveira Junior

**Affiliations:** 1 Centro de Ciências Naturais e Humanas, Universidade Federal do ABC, Santo André, SP 09210580 Brazil; 2 Departamento de Bioquímica, Universidade Federal de São Paulo, São Paulo, SP 04044020 Brazil; 3 Machine Biology Group, Departments of Psychiatry and Microbiology, Institute for Biomedical Informatics, Institute for Translational Medicine and Therapeutics, Perelman School of Medicine, University of Pennsylvania, Philadelphia, PA USA; 4 Departments of Bioengineering and Chemical and Biomolecular Engineering, School of Engineering and Applied Science, University of Pennsylvania, Philadelphia, PA USA; 5 Penn Institute for Computational Science, University of Pennsylvania, Philadelphia, PA USA; 6 Departamento de Biofísica, Universidade Federal de São Paulo, São Paulo, SP 04044020 Brazil; 7 Laboratório de Toxinologia Aplicada (LETA) - Instituto Butantan, São Paulo, SP 05503900 Brazil

**Keywords:** Antimicrobials, Microbiology

## Abstract

The physicochemical and structural properties of antimicrobial peptides (AMPs) determine their mechanism of action and biological function. However, the development of AMPs as therapeutic drugs has been traditionally limited by their toxicity for human cells. Tuning the physicochemical properties of such molecules may abolish toxicity and yield synthetic molecules displaying optimal safety profiles and enhanced antimicrobial activity. Here, natural peptides were modified to improve their activity by the hybridization of sequences from two different active peptide sequences. Hybrid AMPs (hAMPs) were generated by combining the amphipathic faces of the highly toxic peptide VmCT1, derived from scorpion venom, with parts of four other naturally occurring peptides having high antimicrobial activity and low toxicity against human cells. This strategy led to the design of seven synthetic bioactive variants, all of which preserved their structure and presented increased antimicrobial activity (3.1–128 μmol L^−1^). Five of the peptides (three being hAMPs) presented high antiplasmodial at 0.8 μmol L^−1^, and virtually no undesired toxic effects against red blood cells. In sum, we demonstrate that peptide hybridization is an effective strategy for redirecting biological activity to generate novel bioactive molecules with desired properties.

## Abstract (web summary)

A hybrid antimicrobial peptide (AMP) design derived from scorpion venom and parts of other naturally occurring AMPs leads to AMPs with increased antimicrobial activity and minimal toxicity in a mouse infection model and red blood cells.

## Introduction

The COVID-19 pandemic has made it apparent that infectious diseases are an ongoing threat. These diseases are caused by a variety of infectious agents including bacteria and eukaryotic parasites, as well as viruses, constituting leading causes of mortality worldwide. The widespread increase in antimicrobial-resistant bacterial infections is predicted to soon become a leading cause of morbidity and mortality in the world^1^. The global burden of antimicrobial resistance is already well recognized, with a predicted number of deaths per year surpassing 1.27 million^2^. Parasitic diseases, such as malaria, are also lethal, particularly in Africa, Asia, South America, and Central America. The World Health Organization (WHO) has estimated that malaria caused more than 409,000 deaths worldwide in 2019 alone^3^. Therefore, new strategies to treat infectious diseases are urgently needed. Peptides represent promising candidates^4–6^.

The biological activity of peptides can be harnessed for the development of antimicrobial drugs. Antimicrobial peptides (AMPs) are versatile molecules that present diverse mechanisms of action against pathogenic microorganisms, with the added advantage that, unlike conventional antibiotics, they do not readily select for bacterial resistance^7,8^. The activity of AMPs against eukaryotic cell types, such as parasites^9^ and cancer cells^10^, is being explored because the physicochemical and structural features needed to exert these effects are tunable. These features can be enhanced by rational design, machine learning, and peptide engineering^11–14^.

Most AMPs are cationic, small, and amphipathic^15^ and have broad-spectrum activity against pathogenic microorganisms; some, however, also exhibit cytotoxic activity against mammalian cells^16,17^. They tend to form α-helices upon contact with hydrophilic/hydrophobic interfaces, such as biological membranes, which are composed primarily of phospholipids^14^. Despite their promising activity profiles, several limitations have prevented the translation of naturally occurring AMPs into the clinic^18^, including cytotoxicity toward mammalian cells, instability, immunogenic effects, and high manufacturing costs^13^. In recent years, several approaches have been developed to optimize the design of synthetic peptides^14^ with improved biological activities and bioavailability, including computational methods^12^ such as structure-activity relationship studies, neural networks, genetic and pattern recognition algorithms^7,8,19–21^, and machine learning^22–25^; synthetic libraries^26^; template-assisted methodologies^27^; and sequence mutations^28,29^. The most common techniques exploited for sequence mutations are amino acid substitutions^30,31^ or deletions^32^, design of truncated peptides^33,34^, and hybridization of peptides^35–38^. Hybridization, however, has not been as fully exploited as it might be, as a route to rational peptide design.

Arachnids and insects are rich sources of amphipathic AMPs^39^. Scorpions, for example, have several bioactive molecules in their venom, including AMPs^40^. VmCT1, a linear helical AMP isolated from the venom of the scorpion *Vaejovis mexicanus*, has been reported to display antimicrobial and hemolytic activity^41^. Examples of AMPs obtained from wasp venom with broad-spectrum antimicrobial activity^39^ (defined here as activity against both Gram-positive and Gram-negative bacteria) include anoplin, isolated from the venom of *Anoplius samariensis*^42^; protonectin, isolated from *Agelaia pallipes*, which has been reported as a non-hemolytic AMP;^43^ and decoralin, which is derived from *Oreumenes decorates* and has anti-leishmania activity and low hemolytic activity^44^. Amphibians are also important sources of potent bioactive peptides^45^, and AMPs that present antimicrobial^46^, antiplasmodial^47^, and anticancer activities^48^ have been isolated from frog skin secretions. Temporin, derived from the skin of the European frog *Rana temporaria*, is a well-known AMPs that exhibits antimicrobial activity but minimal hemolytic activity^46,49^.

**Fig. 1:**
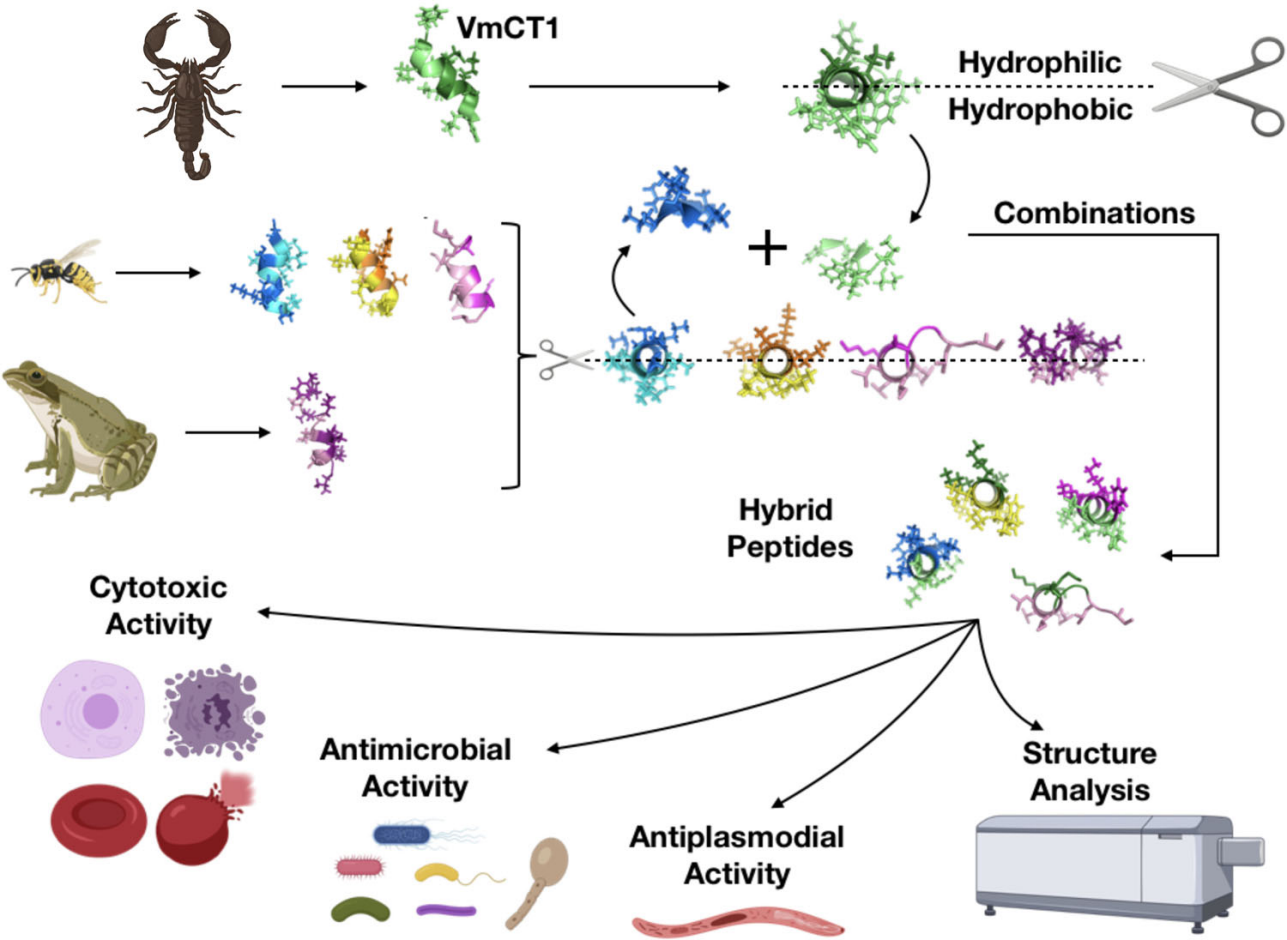
Schematic figure of the design used to generate hybrid peptides and to evaluate their biological activity. The hydrophilic and hydrophobic faces of the helical structure of the AMPs temporin A, protonectin anoplin, and decoralin were combined with the hydrophilic and hydrophobic face of the AMP VmCT1 to generate hybrid peptides. The hybrid peptides were characterized to determine their secondary structure, stability in the presence of proteases and their in vitro and in vivo antimicrobial, hemolytic, and antiplasmodial activities, and for selected AMPs, their anticancer activities.

Although AMPs are represented by diverse peptide families and exert a range of biological activities, they have similar helical structures, lengths, and ratios of polar-to-non-polar residues. Therefore, we selected them as templates for the formation of hybrid peptides. Hybrid peptides are synthetic molecules that contain amino acids or regions from two or more naturally occurring peptide sequences^50,51^. In peptide hybridization, such parts are combined to determine whether their biological activities are altered^52,53^. Here, we combined the hydrophilic and hydrophobic faces of the amphipathic helical structures of anoplin, protonectin, decoralin, and temporin A with portions of the venom peptide VmCT1 to yield seven hybrid peptides. The venom peptide VmCT1 was selected as our design scaffold because of our extensive structure-activity knowledge of this molecule^54^. The hydrophilic and hydrophobic faces of VmCT1 were hybridized with amphipathic helical faces of anoplin, protonectin, decoralin, and temporin A to generate a series of highly active and non-toxic multifunctional peptides (Fig. 1). Peptide hybridization represents a promising molecular rearrangement strategy for optimizing the antimicrobial and antiplasmodial properties of AMPs while minimizing their cytotoxicity.

## Results and discussion

### Design of hybrid antimicrobial peptides

**Fig. 2:**
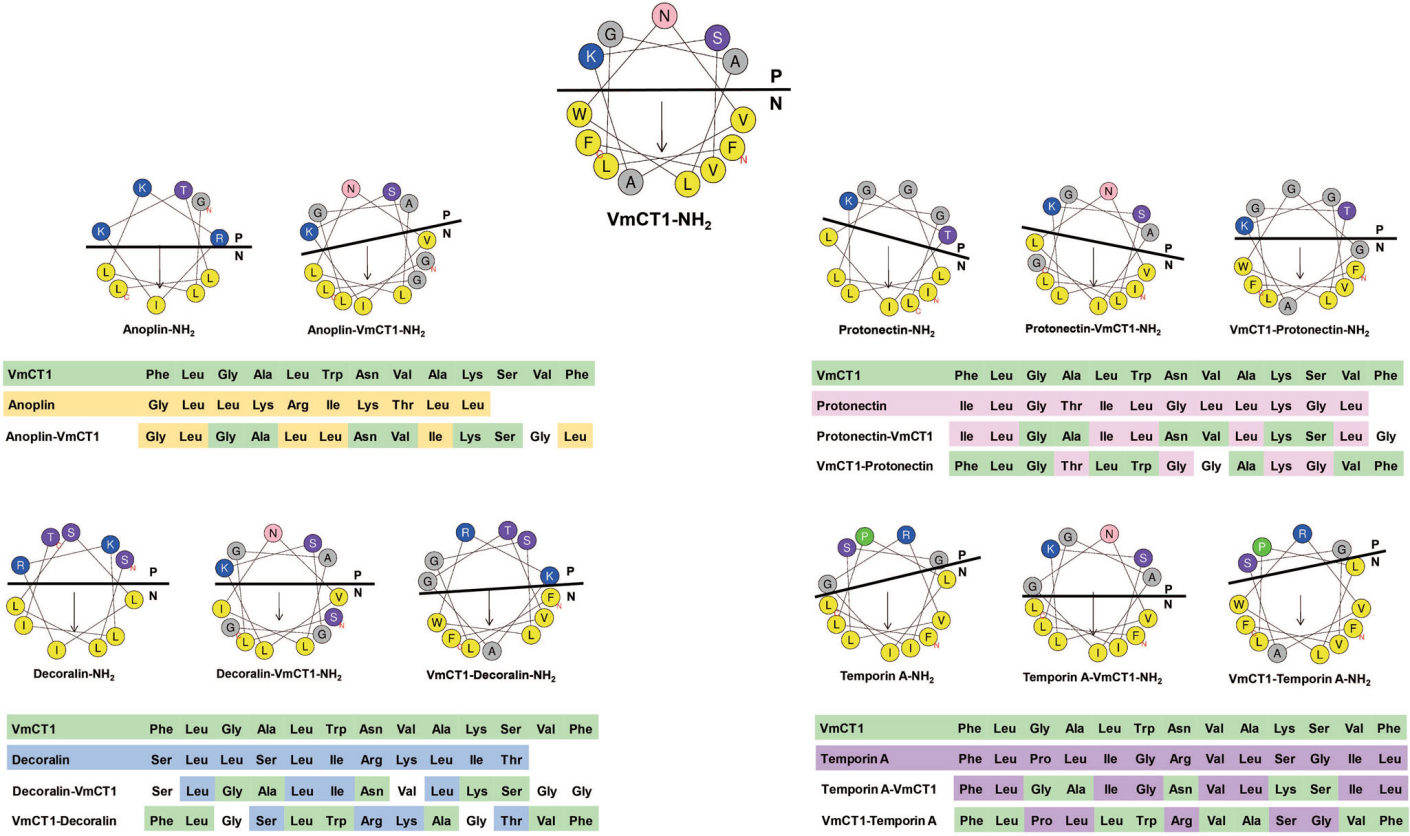
Hybrid peptide design. Helical wheel projections of VmCT1, anoplin, protonectin, decoralin, temporin A, and the hybrid peptides described in this study. The yellow circles indicate aromatic and aliphatic hydrophobic residues; gray circles correspond to residues with hydrophobicity close to zero; blue circles point to basic positively charged residues; purple circles represent polar uncharged residues; green circles represent the restricted pseudo amino acid proline; and pink circles represent polar uncharged amino acid residues. Black arrows indicate the direction and intensity of the hydrophobic moment^84^. P and N indicate the hydrophilic and hydrophobic faces of the amphipathic projection, respectively.

We designed seven hybrid antimicrobial peptides (hAMPs) derived from VmCT1, a highly active broad-spectrum AMP, and four naturally occurring AMP templates and investigated their biological activities. To design the hAMPs, we first used the HeliQuest server to predict the helical wheel projection since all the selected parent AMPs are known to be helical upon contact with membrane bilayers. The server considers a perfect helical disposition (100° between amino acid residues) and projects the sequence in two dimensions. We used those projections to curate which amino acid residues are part of the hydrophilic and hydrophobic faces of the helical structure (Fig. 2). Next, we combined the hydrophilic face of the main parent AMP VmCT1 with the hydrophobic face of one of the other four parent AMPs and vice-versa (Fig. 2 and Table 1). In addition to their helical secondary structure, the template peptides (anoplin, protonectin, decoralin, and temporin A) were also selected based on their amphiphilicity, potent antimicrobial activity, and low hemolytic activity^42–44,46^. These AMPs have slightly different numbers of amino acid residues in their sequences; therefore, we added Gly residues (the simplest side chain group and commonly used as a spacer^55^) to the hydrophobic/hydrophilic interfaces, i.e., the frontier between the hydrophobic and hydrophilic faces in the helical wheel projection for each hAMP, to align sequence sizes in a pairwise manner. Thus, all designed hAMP sequence lengths were standardized to a total of 13 amino acid residues, a size equivalent to that of the template molecule VmCT1.

**Table 1:** Peptides sequence, molecular characterization, and physiochemical properties^84^.

Label	Peptide	Sequence^a^	Molecular weight (Da)		H	µH	z	P/N
			Theoretical	Observed				
**V**	**VmCT1**	**Phe-Leu-Gly-Ala-Leu-Trp-Asn-Val-Ala-Lys-Ser-Val-Phe**	1450.7	1451	0.82	0.58	+2	0.50
A	Anoplin	Gly-Leu-Leu-Lys-Arg-Ile-Lys-Thr-Leu-Leu	1152.5	1153	0.59	0.71	+4	1.00
AV	Anoplin-VmCT1	Gly-Leu-**Gly**-**Ala**-Leu-Leu-**Asn**-**Val**-Ile-**Lys**-**Ser**-Gly-Leu	1253.5	1254	0.65	0.55	+2	0.86
P	Protonectin	Ile-Leu-Gly-Thr-Ile-Leu-Gly-Leu-Leu-Lys-Gly-Leu	1209.6	1210	0.95	0.68	+2	0.71
PV	Protonectin-VmCT1	Ile-Leu-**Gly**-**Ala**-Ile-Leu-**Asn**-**Val**-Leu-**Lys**-**Ser**-Leu-Gly	1309.6	1310	0.77	0.66	+2	0.62
VP	VmCT1-Protonectin	**Phe**-**Leu**-**Gly**-Thr-**Leu**-**Trp**-Gly-Gly-**Ala**-Lys-Gly-**Val**-**Phe**	1351.6	1351	0.77	0.52	+2	0.86
D	Decoralin	Ser-Leu-Leu-Ser-Leu-Ile-Arg-Lys-Leu-Ile-Thr	1254.8	1256	0.78	0.65	+3	0.83
DV	Decoralin-VmCT1	Ser-Leu-**Gly**-**Ala**-Leu-Ile-**Asn**-**Val**-Leu-**Lys**-**Ser**-Gly-Gly	1227.4	1229	0.52	0.46	+2	1.16
VD	VmCT1-Decoralin	**Phe**-**Leu**-**Gly**-Ser-**Leu**-**Trp**-Arg-Lys-**Ala**-Gly-Thr-**Val**-**Phe**	1480.7	1481	0.69	0.58	+4	0.86
T	Temporin A	Phe-Leu-Pro-Leu-Ile-Gly-Arg-Val-Leu-Ser-Gly-Ile-Leu	1396.8	1397	1.00	0.61	+2	0.44
TV	Temporin A-VmCT1	Phe-Leu-**Gly**-**Ala**-Ile-Gly-**Asn**-Val-Leu-**Lys**-**Ser**-Ile-Leu	1343.7	1344	0.80	0.71	+2	0.62
VT	VmCT1-Temporin A	**Phe**-**Leu**-Pro-Leu-**Leu**-**Trp**-Arg-**Val**-**Ala**-Ser-Gly-**Val**-**Phe**	1503.8	1504	1.00	0.47	+2	0.30

*H* hydrophobicity, *μH* hydrophobic moment, *z* net charge, *P/N* ratio of polar/non-polar residues in the sequence. Underlines highlight Gly residues that were inserted so all peptides would have matching hydrophobic and hydrophilic faces in the helical wheel projections.

H, μ_H_, z, and P/N according to helical wheel projection server Heliquest^84^.

Mass obtained under the following conditions: Phenomenex Gemini C18column (2.0 mm × 150 mm, 3.0 µm particles, 110 Å pores). Solvent A was 0.1% TFA in water, and solvent B was 90% ACN in solvent A. Elution with a 5–95% B gradient was performed over 20 min, 0.2 mL min^−1^ flow, and peptides were detected at 220 nm. Mass measurements were performed in a positive mode with the following conditions: mass range between 100 and 2500 m/z, ion energy of 5.0 V, nitrogen gas flow of 12.0 L min^−1^, solvent heater of 250 °C, multiplier of 1.0, capillary of 3.0 kV and cone voltage of 35 V.

Amino acid residues highlighted in bold are originally from VmCT1’s sequence.

^a^All sequences present amidated C-terminus.

By choosing this standard size, we avoided the insertion of hydrophobic residues from the template peptides (anoplin, protonectin, decoralin, and temporin) into the hydrophilic face of VmCT1, as well as the need to insert hydrophilic residues from the other peptides into the hydrophobic face of VmCT1 (Fig. 2). The designed hybrid peptides were designated as: AV, VP, PV, VD, DV, VT, and TV (A is anoplin; V is VmCT1; P is protonectin; D is decoralin; and T is temporin A). For each hybrid peptide acronym, the first letter refers to the hydrophilic face whereas the second letter indicates the hydrophobic face.

Not all hybridization combinations could be synthesized using our methodology. For example, we were unable to synthesize peptide VA combining the hydrophilic portion of VmCT1 with the hydrophobic portion of anoplin, although we attempted various solid-phase synthesis and manual synthesis strategies using conditions such as a range of coupling agents [e.g., *N,N,N*′*,N*′-tetramethyl-*O*-(1*H*-benzotriazol-1-yl)uronium hexafluorophosphate, 2-(1H-Benzotriazole-1-yl)-1,1,3,3-tetramethylaminium tetrafluoroborate, hydroxybenzotriazole] and coupling reaction times (e.g., 30–120 min at room temperature).

### Antimicrobial activity of hAMPs

**Fig. 3:**
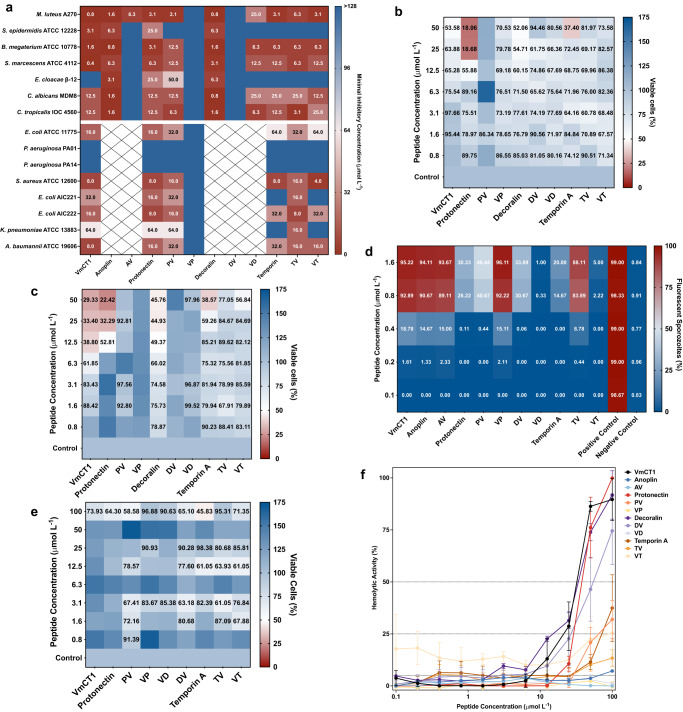
Antimicrobial, antiplasmodial, cytotoxicity, anticancer, and hemolytic activities of hAMPs and their parent AMPs. **a** Peptides in Peptone, Potato Dextrose, or Luria-Bertani broths were exposed to a fixed number of pathogenic (fungi, Gram-positive, and Gram-negative bacteria) cells (10^6^ CFU mL^−1^) in a gradient of concentrations (0–64 μmol L^−1^). Medium with no peptides and no bacteria was used as the control for microbial growth. Absorbance values colored in red represent maximal inhibition of microbial growth. On the contrary, blue corresponds to absorbance values reflective of growth (Anoplin-VmCT1: AV, Protonectin-VmVT1: PV, VmCT1-Protonectin: VP, Decoralin-VmCT1: DV, VmCT1-Decoralin: VD, Temporin A-VmCT1: TV, and VmCT1-Temporin A: VT). Anoplin, AV, Decoralin, DV, and VD were not tested against the ESKAPE pathogens. MTT assay results for peptides after (**b**) 4 h (upper left panel) and (**c**) 24 h (upper right panel) using MCF-7 cancer cell lines. Dulbecco’s Modified Eagle Medium was used as medium for the growth of MCF-7 and MCF-10A cell lines. Absorbance values in red are reflective of a decrease in viable MCF-7 cells, whereas blue correspond to cells that preserved viability. Experiments were performed in three independent replicates. **d** Values are expressed as the percentage of fluorescent mature sporozoites. Absorbance values in red represent active compounds against *Plasmodium gallinaceum* (fluorescent sporozoites), whereas absorbance values in blue indicate lack of antiplasmodial activity. Digitonin/PBS solution was used as the positive control and PBS solution alone was used as the negative control. **e** MTT assay results for healthy human breast epithelial cells MCF-10A lines exposed to the peptides for 24 h. Dulbecco’s Modified Eagle Medium was used as the control for cell maximum growth. Again, in these experiments, blue represented viable MCF-10A cells, whereas red corresponded to cell death. Experiments were performed in three independent replicates. **f** Red blood cells were exposed for 1 h and at room temperature to peptides at concentrations ranging from 0.1 to 100 μmol L^−1^, in PBS. Surfactant (1% SDS in PBS) was used to ensure complete hemolysis and PBS was used as a control to preserve erythrocyte integrity. A PBS solution and the surfactant SDS were used as negative and positive controls, respectively. The MHC (maximal non-hemolytic concentration) was determined as the maximal concentration at which 100% of the erythrocytes were viable. All assays were done in three independent replicates. The graph in **f** shows mean and standard deviation.

The template peptides (VmCT1, anoplin, protonectin, decoralin, and temporin A) exhibited MICs against bacterial strains ranging from 0.8 to 25 μmol L^−1^ (Fig. 3a and Tables S1 and S2). Of the seven hybrid peptides tested in this study, those with the highest antibacterial activity (i.e., lowest MICs) were TV and VT: 3.1 to 25 μmol L^−1^ (Fig. 3a and Tables S1, S2). TV presented slightly more potent activity against Gram-negative strains (Fig. 3 and Tables S1 and S2). The next highest antimicrobial activity was observed with PV (3.1–64 μmol L^−1^) and VD (6.3–25 μmol L^−1^). In most cases, the range of antimicrobial activity of the hybrid peptide matched, or came within one dilution, of that of the template AMP (protonecin and temporin A, 3.1–25 μmol L^−1^). AV and VP, as well as one of the two hybrid peptides derived from decoralin (VD), had lower antimicrobial activities than their corresponding template peptides. The antimicrobial activity (MICs) of AV ranged from 6.3 to 50 μmol L^−1^, whereas the MICs of anoplin (Fig. 3a and Tables S1 and S2) ranged from 0.8 to 6.3 μmol L^−1^. No antimicrobial activities were observed with DV. The MIC values of protonectin, PV and VP against *P. aeruginosa* were 128 μmol L^−1^ (Fig. 3a and Tables S1 and S2). Anoplin and protonectin were not active against *Bacillus subtilis* at the range of concentrations tested. AV and VP, as well as the two hybrid peptides derived from decoralin (VD and DV), had lower antimicrobial activities than the corresponding template peptides.

### Antiplasmodial activity of hAMPs

We then assessed the activity of the hybrid peptides against *P. gallinaceum*, which is the closest model to *Plasmodium falciparum* infection available with no risk of human infection^56^, using fluorescence microscopy, as previously described^57,58^. The fluorescent *P. gallinaceum* sporozoites are those whose genetic material has been stained by propidium iodide, denoting a damaged membrane (Fig. 3d and Table S1). In this assay, the parent peptides decoralin and temporin A showed no activity. AV, on the other hand, was as active against *P. gallinaceum* sporozoites as its parent peptides anoplin and VmCT1: 0.8 μmol L^−1^ of AV and anoplin yielded 90% fluorescent sporozoites, and VmCT1 93% fluorescent sporozoites. VP and TV also displayed antiplasmodial activity: 92% and 83% of the sporozoites were killed by these hybrid peptides, respectively, at 0.8 μmol L^−1^ (Fig. 3d and Table S1). VD, DV, and VT did not exhibit significant antiplasmodial activity compared to the other molecules designed in this work at the concentration range tested.

### Activity of hAMPs on healthy and cancer human cell lines

In comparison with healthy cell membranes, cancerous cell membranes typically present higher levels of anionic molecules, such as phosphatidylserines, glycoproteins with net negative charge, and glycosaminoglycans, favoring electrostatic interactions between cationic peptides and the cell membrane^59–61^. Since the design principles used here led to peptides with activity against negatively charged cell membranes, we assessed the activity of the hybrid peptides (except for anoplin and AV) against MCF-7 human breast cancer cells after 4 and 24 h of incubation (Fig. 3b, c and Table S1).

VD, unlike the other hybrid peptides, had selective activity against MCF-7 human breast cancer cells at 25 μmol L^−1^. However, VD showed decreased activity compared to its template peptides VmCT1 and decoralin (50 μmol L^−1^). Within 4 h of incubation with MCF-7, protonectin and temporin A reduced the counts of viable MCF-7 cells (LD_50_: 25 μmol L^−1^ and 50 μmol L^−1^, respectively). After 24 h of incubation, protonectin and temporin A exhibited the same LD_50_ (LD_50_: 25 μmol L^−1^ and 50 μmol L^−1^, respectively). As a control, our designed peptides were also tested against MCF-10A healthy human breast epithelial cells (Fig. 3e). After 24 h of incubation, we did not observe peptide-meditated cytotoxicity towards MCF-10A cells (Fig. 3e).

### Hemolytic activity of hAMPs

Hemolytic activity is a key measure of potential toxicity or safety for human cells. Hemolytic activity, here defined as the concentration of peptide needed to kill 10% of red blood cells, was observed with the parent peptides decoralin and temporin, which were hemolytic at 1.6 μmol L^−1^ and 0.2 μmol L^−1^, respectively. Anoplin was hemolytic at 50 μmol L^−1^ (Fig. 3f, Table S1, and Supplementary Data 1). DV was significantly hemolytic at low concentrations (0.1 μmol L^−1^), and VT and TV presented hemolytic activity at 0.2 μmol L^−1^ (Fig. 3f, Table S1, and Supplementary Data 1). The hybrid peptides having the lowest hemolytic activity were AV and VP (100 μmol L^−1^) along with VD, for which no hemolytic activity was detected in our assay (Fig. 3f, Table S1, and Supplementary Data 1). The markedly improved hemolytic profile of VP (hemolytic at 100 μmol L^−1^) compared to the parent peptide protonectin (hemolytic at 12.5 μmol L^−1^) or to PV (hemolytic at 25 μmol L^−1^) suggests that peptide hybridization can produce derivatives with improved safety profiles.

### Relationship of structural characteristics to peptide activity

**Fig. 4:**
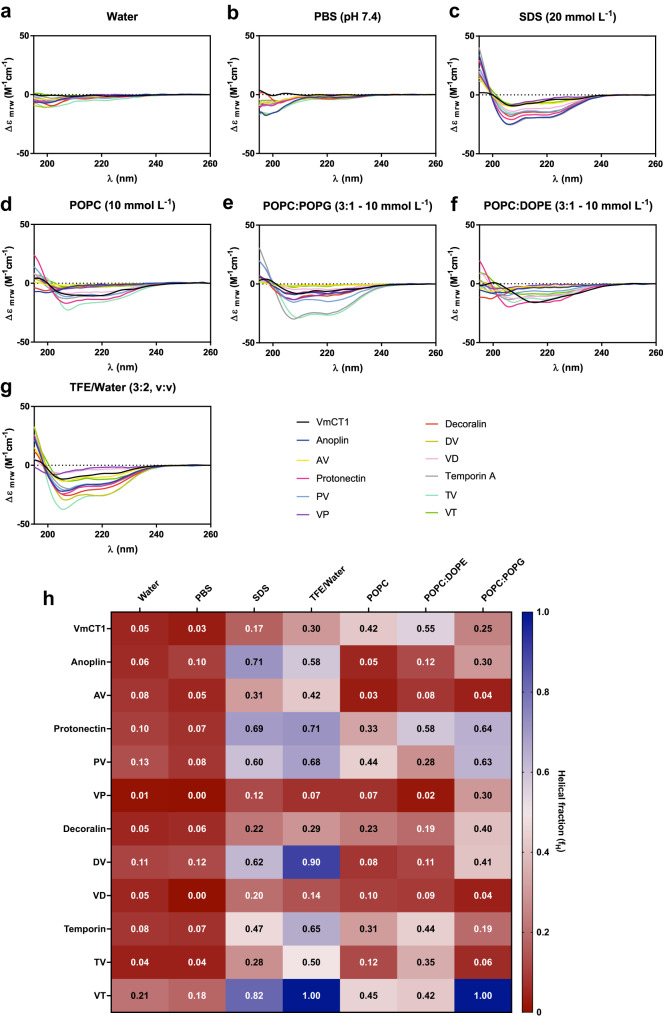
Structural analysis of hAMPs. Circular dichroism (CD) spectra of the parent peptides and the hAMPs designed in this study in (**a**) water, (**b**) PBS (pH 7.4), (**c**) SDS (20 mmol L^−1^), (**d**) POPC (10 mmol L^−1^), (**e**) POPC:POPG (3:1, mol:mol - 10 mmol L^−1^), (**f**) POPC:DOPE (3:1, mol:mol - 10 mmol L^−1^), and (**g**) TFE/Water (3:2, v-v). CD spectra were recorded after four accumulations at 20 °C, using a 1 mm path length quartz cell, between 195 and 260 nm at 100 nm min^−1^, with a band width of 0.5 nm. All assays were performed with peptides at 50 μmol L^−1^. **h** Helical fractions (f_H_) values were calculated based on the Lifson-Roig model for each of the conditions analyzed (**a**–**g**).

To assess whether we could link the observed bioactivities to the structural characteristics of the hybrid peptides, we evaluated their secondary structure by circular dichroism (CD; Fig. 4a–g and Supplementary Data 2–8). Anoplin is an α-helical AMP isolated from *Anoplius samariensis* wasp venom. This peptide is composed of 10 amino acid residues and has an amidated carboxyl-terminus^42^. AV, which has Gly residues in positions 1 and 12, presented a lower net charge (+2) than anoplin itself and had a lower hydrophobic moment than VmCT1 or anoplin (Table 1).

Pedron et al. reported that Lys-substitutions of VmCT1 analogs increased the net positive charge of this family of peptides and were important for their increased antimicrobial activity^54^. Munk et al. synthesized 19 anoplin analogs and found that changes in net positive charge and hydrophobicity-related properties influenced activity against erythrocytes and microorganisms^62^. Therefore, we attribute the lower antimicrobial activity of AV to its lower net positive charge value compared to its parent molecules. The helical content of AV was lower than that of anoplin for all the media analyzed and higher than the helical content of VmCT1 in SDS (f_H_: 0.31, and 0.17, respectively) and TFE/water solution (f_H_: 0.42 and 0.30, respectively) (Fig. 4 and Table S3).

Protonectin, an AMP isolated from *Agelaia pallipes* wasp venom, is composed of 12 amino acid residues and presented hemolytic activity at 12.5 μmol L^−1^, but shows potent antimicrobial activity against bacteria^43^. VP was designed with one additional Gly residue placed at position 8.

The CD assays (Fig. 4a–g) suggested that the active hybrid peptides derived from VmCT1 and protonectin presented high (>60%) helical content. VP exhibited low helical content values in all the media tested, whereas PV presented higher helical content than VmCT1 in all media used, with helical fraction values higher than 0.5, e.g., SDS (f_H_: 0.60) POPC: POPG (f_H_: 0.63) and TFE/water solution (f_H_: 0.68) (Fig. 4c, e, g, respectively, and Fig. 4h and Table S3) and higher helical content than protonectin in POPC (f_H_: 0.44 and 0.33, respectively) (Fig. 4d, h and Table S3).

Protonectin displayed homology with polybia-CP, though it differs at position 11, where protonectin has a Gly residue and polybia-CP contains a Ser residue. Torres et al. described an Alanine-scan screening of polybia-CP and verified that modifications within the hydrophobic face of the peptide decreased its antimicrobial activity, revealing that its hydrophobic face was crucial for antimicrobial activity^28^. These observations are consistent with our results for PV, which incorporated protonectin’s hydrophobic face (Fig. 2) displaying increased antimicrobial activity than VP (Fig. 3). Additionally, VP exhibited high antiplasmodial activity (92% of killed sporozoites) at 0.8 μmol L^−1^ (Fig. 3b and Table S1). In contrast, PV presented lower antiplasmodial activity at the same concentration killing only 41% of the sporozoites (Fig. 3d and Table S1). The antiplasmodial activity observed for this peptide confirms that its helical content is less important to its antiplasmodial activity than to its antimicrobial and cytotoxic activities (Fig. 3a–c, e and Table S1). These results are in line with previous observations by Torres et al., who showed that the anti-*Plasmodium* activity of small cationic decoralin analogs did not depend on their α-helical structure^57^. The authors showed that decoralin’s N-terminal extremity motif was crucial for its antiplasmodial activity. Decoralin, an AMP isolated from the venom of the *Oreumenes decoratus* wasp and containing 11 amino acid residues and an amidated C-terminal extremity, tends to adopt a helical structure and has broad-spectrum antimicrobial activity and low hemolytic activity at 100 µmol L^−1^ ^44^.

Peptide VD, which had two Gly residues as spacers in positions 3 and 10, presented low helical content in all media tested. On the contrary, DV exhibited higher helical content than VmCT1 in all media used (Fig. 4a–h and Table S3). No effect was found between a low helical content and hemolytic or antimicrobial activities. DV, which also had two additional Gly residues at positions 12 and 13, did not exhibit antimicrobial activity at the range of concentrations tested and was significantly hemolytic even at low concentrations (0.1 μmol L^−1^). Torres et al.^63^ similarly described Leu-substituted decoralin analogs, in which the peptide with a Leu-substitution at position 8 presented lower helical content in TFE/water solution (f_H_: 0.24) than decoralin and had increased antimicrobial activity and lower hemolytic activity with a minimal hemolytic concentration (MHC) value of 50 μmol L^−1^ (Table S1), showing that changes in conformational tendencies affected both antimicrobial and hemolytic activities. Thus, helical structure was not found to be a determinant of antimicrobial activity for hybrid peptides derived from VmCT1 or decoralin.

The antiplasmodial activity of decoralin analogs was previously described by Torres et al.^57^. Although decoralin itself did not present antiplasmodial activity, rationally designed synthetic decoralin analogs had antiplasmodial activity at 60 μmol L^−1^, i.e., a concentration higher than that of the hybrid peptides designed in the present study. Like the native decoralin, VD and DV did not present antiplasmodial activity (Fig. 3d and Table S1).

The temporins are a group of peptides with 10 to 13 amino acid residues in their sequence^46,49^. Temporin A exhibits activity against Gram-positive and Gram-negative bacteria and low hemolytic activity^46^, which made it a promising candidate for hybridization with VmCT1. VT and TV presented higher helical content values than VmCT1. VT also presented higher helical content than temporin A, but TV had lower helical content values than temporin A in all media tested (Fig. 4h and Table S3), the increased observed helicity of the hybrids did not lead to increased bioactivity compared to the templates (Fig. 4h).

### hAMPs resistance to proteolytic degradation

**Fig. 5:**
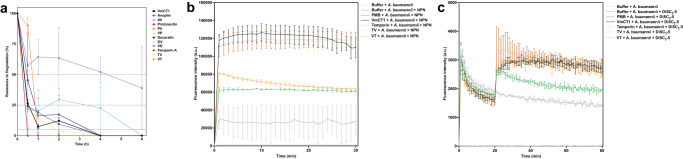
Resistance to enzymatic degradation and mechanism of action of hAMPs. **a** Peptides were exposed to fetal bovine serum for 6 h. Aliquots were collected and analyzed by liquid chromatography. Three independent replicates were performed. **b** Damage and permeabilization of the bacterial outer membrane by the peptides using the NPN assay. **c** Effect of the peptides on the depolarization of the cytoplasmic membrane using the DiSC_3_(5) assay. The graphs in **a**–**c** show both the mean and standard deviation.

The resistance to proteolytic degradation of the hybrid peptides was assessed by exposing them for 6 h to serum peptidases in a fetal bovine serum solution^64,65^ (Fig. 5a, Table S4, and Supplementary Data 9). The peptide degradation kinetics were calculated by integrating the area under the curve of peaks relative to each peptide at the beginning of the experiment (time = 0) and comparing it to the integrated peak area relative to the peptides after exposure to the enzymes at time points: 0.5, 1, 2, 4, and 6 h (Fig. 5a, Table S4, and Supplementary Data 9). VmCT1 was quickly degraded, with ~20% of peptide remaining after 30 min of exposure to serum proteases and <10% after 1 h (Fig. 5a, Table S4, and Supplementary Data 9). Anoplin was slightly more stable: less than 50% of peptide remained after 30 min of exposure to peptidases. However, AV was resistant to degradation against proteases, with almost 40% peptide content remaining after 4 h of exposure (Fig. 5a, Table S4, and Supplementary Data 9). Protonectin was quickly degraded (Fig. 5a, Table S4, and Supplementary Data 9), whereas PV resisted for 30 min (~90% of peptide remaining); however, it was totally degraded after 1 h of exposure. VP was almost entirely degraded after 30 min (22% of peptide remaining). Temporin A, TV, and VT were totally degraded within 30 min after the start of the experiment (Fig. 5a, Table S4, and Supplementary Data 9). D-amino acid substitutions have been used as alternatives to prevent the degradation of AMPs. For example, Qiu et al. described a protonectin analog with a _D_-Lys residue in position 10 as an alternative to protonectin; this analog is not degraded and has higher antimicrobial activity against bacteria and fungi than the original peptide. In addition, the authors showed that _D_-protonectin was stable in the presence of trypsin, chymotrypsin, and human serum, whereas d-Lys-protonectin showed stability only against trypsin^66^.

The most resistant peptide among our synthesized analogs was VD, the decoralin-derived hAMP, which has a Trp residue in position 6. VD resisted degradation (~50% of peptide remaining) for 6 h (Fig. 5a, Table S4, and Supplementary Data 9). Torres et al. reported the importance of position 6 in the decoralin sequence to the resistance to peptidases^67^. The authors verified that substituting position 6 (Ile) by a Phe residue created analogs with higher resistance to degradation^67^.

### Mechanism of action of hAMPs

To assess the disruptive and permeabilizing effect of the most active hAMPs (TV and VT) and their parent peptides VmCT1 and temporin A on the outer membrane of *A. baumannii*, we used the probe 1-(N-phenylamino)-naphthalene (NPN), as previously described^68,69^. The peptides VmCT1, temporin A, and TV increased fluorescence compared to the control, indicating the permeabilization of *A. baumannii* cell membranes. On the contrary, the hybrid peptide VT did not lead to increased fluorescence compared to the control (Fig. 5b and Supplementary Data 10).

Membrane depolarization constitutes another mechanism of antibacterial activity. To study whether the hybrid peptides derived from temporin A depolarized cytoplasmic membranes, we used the probe 3,3′-dipropylthiadicarbocyanine iodide [DiSC_3_(5)]^68,69^. The hybrid peptides tested, VT and TV, depolarized the cytoplasmic membrane of *A. baumannii*, presenting increased fluorescence compared to the controls (Fig. 5c and Supplementary Data 11).

### Skin abscess infection mouse model and histopathological assays

**Fig. 6:**
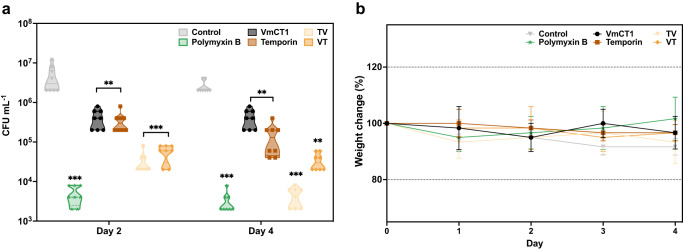
Anti-infective activity in a skin abscess infection mouse model. **a** Anti-infective activity of VmCT1, Temporin A, VT, and TV peptides in a mouse model of *A. baumannii* skin infection as detailed in the methodology section (*n* = 8). Statistical significance was determined using two-way ANOVA followed by Dunnett’s test, **p < 0.01 and ***p < 0.001. **b** The toxicity of the peptide was monitored by mouse body weight measurements obtained during the experiment. Mouse weight changes were normalized to the body weight of each mouse at the beginning of the experiment, as a proxy for potential toxicity. The graph in **b** shows mean and standard deviation.

The most potent peptides described in this study (Fig. 6a and Supplementary Data 12), VmCT1, temporin A, VT, and TV, were evaluated for their anti-infective activity in a skin infection mouse model^28,69,70^ against *A. baumannii*, which is responsible for nosocomial and secondary infections with high death rates^71^. The skin infection was made by a superficial abrasion on the back of the mouse, which injures the stratum corneum and the upper layer of the epidermis. *A. baumannii* (10^7^ CFU) was inoculated at the scarification site. As a proxy for toxicity, we monitored the weight and visible changes, such as redness, lethargy, morbidity, and loss of fur, over the course of the experiment. Two days post-treatment, TV and VT showed increased anti-infective activity when compared to the parent peptides (VmCT1 and temporin A) and results that are statistically comparable to those obtained with the conventional antibiotic polymyxin B (PMB). Four days after the treatment, both hybrid peptides TV demonstrated significant anti-infective activity comparable to PMB, reducing the load of *A. baumannii* cells in the infected area by two orders of magnitude (Fig. 6a and Supplementary Data 12) and VT presented the same effect as the two parent peptides. Peptide treatment did not lead to significant changes in weight (Fig. 6b and Supplementary Data 13) or other visible side effects.

Next, we selected the most active hybrid peptide (TV) and its parent VmCT1 to assess their histopathological effect on skin tissue. First, we performed the skin abscess infection mouse model and euthanized the mice and collected their tissue 24 h after infection and treatment. We used hematoxylin and eosin (H&E) staining to stain the nuclei and extracellular matrix and cytoplasm, respectively, followed by optical microscopy (Figure S1). Neither TV nor VmCT1 changed the tissue’s morphology compared to the control polymyxin B and the infected untreated or uninfected samples. Thus, we further confirmed that the peptides studied here did not display toxicity.

## Conclusions

In this study, we designed a series of hybrid peptides combining the hydrophobic and hydrophilic faces of the amphipathic helical structure of the venom peptide VmCT1 with parts of other active AMPs (i.e., anoplin, protonectin, decoralin, and temporin A) of different sizes and physicochemical and structural properties. Our results show that hybridization of the amphipathic regions of AMPs can be explored as a tool for generating new peptides and that some of these may exhibit broad-spectrum activity, anti-infective activity, and lower cytotoxicity for human cells, compared to the original peptides. Our results also indicate that peptide hybridization is useful for analyzing whether hydrophilic and hydrophobic faces contribute to the overall biological activity of the hybrid peptides and potentially offer a guide for constructing AMPs with improved activity profiles.

The hybrid peptides were also tested for their resistance to enzymatic degradation and mechanisms of action. Among the hybrid peptides, VD exhibited the greatest resistance to proteolytic degradation. Our investigation of the mechanism of action showed that the hybrid peptides TV and VT, similar to their parent peptides VmCT1 and temporin A, either disrupted or depolarized the bacterial membrane and revealed their multifunctional mechanism of action. We envision that by rearranging the amphipathic regions of peptides we may be able to create hybrids that overcome activity restrictions and instability in the presence of proteases, which currently limit the application of AMPs in the clinic.

In addition to investigating the antibacterial activity of the hybrid peptides, we looked at their interactions with eukaryotic cells. Several small cationic peptides (AV, VP, and TV) exhibited antiplasmodial activity; our study revealed that the helical content was not an important feature for this activity. Our approach also enabled the generation of several hAMPs with minimal hemolytic activity (e.g., AV, VP, and VD). Although the strategy we used in this study, merging hydrophilic and hydrophobic portions of natural AMPs, was not consistently effective for creating anticancer peptides (e.g., VD), none of the designed peptides exhibited cytotoxic activity against MCF-10A healthy human breast epithelial cells (Fig. 3e).

Finally, two of the hybrid peptides were tested in a mouse model of skin infection. In this skin scarification mouse model, VT and TV had anti-infective activity more potent than that of either parent peptides, VmCT1 and temporin A.

In sum, we demonstrate that the hybridization of amphipathic portions of AMPs is a useful design strategy for generating peptides with broad-spectrum activity at low micromolar concentrations and minimal hemolytic and cytotoxic activities toward human cells. The hybridization of portions of known peptides is an underexplored molecular engineering tool that opens new avenues for the design of bioactive peptides.

## Methods

### Solid-phase peptide synthesis, purification, and characterization

All peptides were synthesized using solid-phase peptide synthesis (PS3-Sync Technologies) and fluoromethyloxycarbonyl (Fmoc) as described by Torres et al.^67^. Crude peptides were lyophilized and subsequently purified by semi-preparative reverse-phase high-performance liquid chromatography (RP-HPLC) on a Delta Prep 600 (Waters Associates). Selected fractions containing the purified peptides were pooled and lyophilized. The characterization steps were performed by liquid-chromatography electrospray-ionization mass spectrometry (LC/ESI-MS) using a Model 6130 Infinity mass spectrometer coupled to a Model 1260 HPLC system (Agilent), following previously described protocols^67^ (Figs. S2–S13). The peptide content was determined by spectrophotometry (Nanodrop™ 2000, Thermo Scientific). To measure the percentage of peptide in the lyophilized sample, the peptides were dissolved in deionized water at a concentration of 1 mg mL^−1^ and the absorbance was measured at 205 and 260 nm. The percentage of the peptide in the sample was determined by the rate between the expected concentration and the concentration determined experimentally^72–74^.

### Microorganisms

Peptides were tested in antimicrobial assays against Serratia marcescens ATCC 4112, Enterobacter cloacae β-12, Micrococcus luteus A270, Bacillus megaterium ATCC 10778, Bacillus subtilis ATCC 6633, Candida albicans MDM8, Candida tropicalis IOC 4560, Acinetobacter baumannii ATCC 19606, Escherichia coli ATCC 11775, Escherichia coli AIC221, Escherichia coli AIC222, Klebsiella pneumoniae ATCC 13883, Staphylococcus aureus ATCC 12600, Pseudomonas aeruginosa PAO1, and Pseudomonas aeruginosa PA14. All microorganisms were obtained from the American Type Culture Collection (ATCC), Instituto Butantan, São Paulo, Brazil, and the clinical isolate strains were donated from the Oswaldo Cruz Institute Collection (IOC), Rio de Janeiro, Brazil.

### Antimicrobial activity

Initially, the antimicrobial activity was determined by liquid growth inhibition assays, as previously described by Pedron et al.^54^. Briefly, Peptone Broth and Potato Dextrose Broth (Invitrogen) were used for antibacterial and antifungal assays, respectively. Two-fold serial dilutions of peptides (0.09–50 μmol L^−1^) and microorganisms (*Serratia marcescens* ATCC 4112, *Enterobacter cloacae* β-12, *Micrococcus luteus* A270, *Bacillus megaterium* ATCC 10778, *Bacillus subtilis* ATCC 6633, *Candida albicans* MDM8, and *Candida tropicalis* IOC 4560) were incubated at 37 ^o^C for 18 h (for bacteria) or 24 h (for fungi), and microbial growth was assessed by absorbance measurements at 595 nm. Experiments were performed in three biological repeats.

The minimal inhibitory concentration (MIC) against the ESKAPE pathogens was determined using the broth microdilution method^7^ in Luria-Bertani (LB) medium. The MIC value was defined as the lowest concentration of peptide at which no microbial growth was observed. *A. baumannii* ATCC 19606, *E. coli* ATCC 11775, *E. coli* AIC221, *E. coli* AIC222, *K. pneumoniae* ATCC 13883, *S. aureus* ATCC 12600 *P. aeruginosa* PAO1, and *P. aeruginosa* PA14 were incubated on agar plates, and after 24 h, three isolated colonies were inoculated to 5 mL of Luria-Bertani (LB) broth and maintained at 37 °C overnight with shaking. Peptides were then added to 96-well polystyrene microtiter plates at concentrations ranging from 0 to 128 μmol L^−1^, and 5 × 10^5^ CFU mL^−1^ of each bacterium was inoculated in each well. The plates were incubated at 37 °C for 24 h. Bacterial growth was measured using a microplate reader at 610 nm. Three independent replicates were performed.

### Antiplasmodial activity against *Plasmodium gallinaceum* sporozoites

The activity of the peptides against *Plasmodium gallinaceum* sporozoites was determined by fluorescence microscopy, as previously described by Silva et al.^75^. Briefly, 9000 sporozoites were isolated from the glands of infected *Aedes aegypti* mosquitos and incubated with serially diluted peptides (ranging from 0.1 to 1.6 μmol L^−1^) at 37 °C for 1 h. A PBS solution and the surfactant digitonin^76,77^ were used as negative and positive controls, respectively. Propidium iodide was used to stain dead cells. Assays were executed using fluorescence microscopy. Three biological repeats each with three different mosquito batches (*n* = 9) were used.

### Cytotoxicity against healthy and cancer human cell lines

The anticancer activity assays were performed using MCF-10A human breast epithelial cells and MCF-7 mammary cancer cells as previously described by Pedron et al.^78^. Briefly, the day before treatment, the cells were incubated at 37 °C and 5% CO_2_ for 24 h. The cells were treated with peptides ranging from 12.5 to 100 μmol L^−1^ (MCF10-A cells) and 0.8 to 100 μmol L^−1^ (MCF-7 cells) for 4 h and 24 h at 37 °C, and cell viability was measured using 3-(4,5-Dimethylthiazol-2-yl)-2,5-diphenyltetrazolium bromide (MTT) assays^79,80^. Three independent replicates were performed in each case.

### Hemolytic activity

Assays against freshly collected human erythrocytes were performed following protocols described by Pedron et al.^54^. Briefly, peptide aliquots at concentrations ranging from 0.1 to 100 μmol L^−1^ were incubated with a suspension of erythrocytes at room temperature for 1 h. The samples were then centrifuged, and the absorbance of the supernatant was measured at 405 nm. The MHC (maximal non-hemolytic concentration) was determined as the maximal concentration at which 100% of the erythrocytes were viable. The surfactant SDS and PBS buffer were used as positive^81,82^ and negative controls, respectively. Sodium heparin was used as an anticoagulant when collecting human blood. Three biological repeats were performed. All experiments were performed in accordance with the protocol CEUA/IB #I-1345/15 approved by the Instituto Butantan.

### Circular dichroism spectroscopy

Circular dichroism (CD) assays were performed with a Spectropolarimeter Jasco Mod. J-815 (JascoCorp). CD spectra were obtained in Far-UV (195–260 nm), and analyzed in the following solutions: water, 2,2,2-trifluoroethanol (TFE) in water (3:2, v-v), phosphate buffer saline (PBS, 10 mmol L^−1^, pH 7.4), sodium dodecyl sulfate (SDS 20 mmol L^−1^) in water, palmitoyloleoylphosphatidylcholine (POPC; 10 mmol L^−1^), palmitoyloleoylphosphatidylcholine: dioleylphosphatidylethanolamine (POPC:DOPE; 3:1, mol:mol, 10 mmol L^−1^) and palmitoyloleoylphosphatidylcholine: palmitoyloleoylphosphatidylglycerol (POPC:POPG; 3:1, mol:mol, 10 mmol L^−1^) at a peptide concentration of 50 µmol L^−1^. These experiments were performed as described in detail by Pedron et al.^54^. Briefly, CD spectra were recorded after four accumulations at 20 °C, using a 1 mm path length quartz cell, between 195 and 260 nm at 100 nm min^−1^, with a band width of 0.5 nm. All assays were performed with peptides at 50 μmol L^−1^.

### Outer membrane permeabilization

Membrane permeabilization was assessed for two of the naturally occurring peptides (VmCT1 and temporin A) and two of the hybrids [VmCT1-temporin A (VT) and temporin A-VmCT1 (TV)] at their MIC by using the *N*-phenyl-1-naphthylamine (NPN) uptake assay as previously described^7^. Briefly, *A. baumannii* ATCC 19606 cells were grown to an optical density of 0.4 at 600 nm (OD_600_), centrifuged (10,000 rpm at 4 °C to 10 min), washed, and resuspended in buffer (5 mmol L^−1^ HEPES, 5 mmol L^−1^ glucose, pH 7.4). Four μL of NPN solution (0.5 mmol L^−1^; working concentration of 10 μmol L^−1^, after dilutions) was added to 100 μL of the bacterial solution in 96-well plates. The background fluorescence was then determined at *λ*_ex_ = 350 nm and *λ*_ex_ = 420 nm. The peptides resuspended in water (100 μL at a concentration of 1.2 μmol L^−1^) were added to each 96-well plate. The fluorescence was determined as a function of time until no further increase in fluorescence was observed (45 min). Three independent replicates were performed in each case.

### Cytoplasmic membrane depolarization

The cytoplasmatic membrane depolarization activity of two of the naturally occurring peptides (VmCT1 and temporin A) and two of the hybrids (VT and TV) was determined at their MIC by performing the membrane potential-sensitive dye DiSC_3_(5), as reported previously^7^. *A. baumannii* ATCC 19606 cells were grown at 37 °C with agitation to an optical density of 0.5 at 600 nm (OD_600_), centrifuged, washed twice with buffer (5 mmol L^−1^ HEPES, 20 mmol L^−1^ glucose, pH 7.2), and resuspended to an OD_600_ of 0.05 in the same buffer but containing 0.1 mol L^−1^ KCl. Cells (100 μL) were incubated for 15 min with 20 nmol L^−1^ of DiSC_3_(5) until a stable reduction in fluorescence was observed, indicating DiSC_3_(5) incorporation into the bacterial membrane. After adding the peptides (100 μL solution at 1.2 μmol L^−1^), membrane depolarization was determined by the change observed in the fluorescence emission intensity of the dye at *λ*_ex_ = 622 nm and *λ*_ex_ = 670 nm. Three independent replicates were performed in each case.

### Resistance to proteolytic degradation

Stability assays were performed following the protocol described by Torres et al.^67^. Briefly, a peptide solution was added to GIBCO fetal bovine serum diluted to 25% in water and kept at 37 °C. The degradation kinetics were monitored by liquid chromatography and the percentage of peptide remaining in each case was calculated by integrating the peptide peak area. These experiments were performed as three independent replicates, and aliquots were taken at 0, 0.5, 1, 2, 4, and 6 h.

### Skin abscess infection mouse model

The anti-infective activity of the peptides VmCT1, temporin A, VT, and TV against *A. baumannii* ATCC 19606 was evaluated as described by Torres et al.^7,83^ Bacteria were grown in tryptic soy broth medium to an optical density of 0.5 at 600 nm. Cells were washed twice with sterile phosphate-buffered saline (pH 7.4, 13,000 rpm for 1 min) and resuspended to a final concentration of 1 × 10^7^ CFU/20 μL. To generate the skin infection, female CD-1 mice (6-weeks-old) were first anesthetized with isoflurane and their backs were shaved. Using a needle, a superficial linear abrasion was made on the shaved area of the mice to injure just the stratum corneum and the upper layer of the epidermis. An aliquot of 20 μL containing 1 × 10^7^ CFU of *A. baumannii* was then inoculated over the wound area with a pipette tip. The next day, the infected area was treated with the peptides (i.e., VmCT1, temporin A, VT, and TV), which were administered onto the abscess at their MIC. The animals were euthanized, and the scarified skin area removed 2- and 4-days post-infection. The excised area was homogenized using a bead beater for 20 min (25 Hz) and serially diluted for CFU quantification. Eight mice were used per group (*n* = 8). All experiments were performed in accordance with protocol 806763 approved by the IACUC at the University of Pennsylvania.

### Skin tissue histopathological analysis

The histopathological effect of the parent peptide VmCT1 and hAMP TV (the most active hybrid peptide in vivo) was assessed by hematoxylin and eosin (H&E) staining of the skin tissue followed by microscopy analysis. The fur on the back of the mice was removed and the mice were infected and treated as described in the *Skin abscess infection mouse model* section. After 24 h, mice were euthanized for tissue collection. Samples were pre-fixed in individual cassettes, then transferred to a container filled with 70% ethanol (EtOH) for slicing and staining. Next, the samples were placed on glass slides for microscopy analysis. We collected the tissue from three mice per condition analyzed.

### Statistics and reproducibility

All assays were performed in at least three independent replicates and all statistical analyses were performed using GraphPad v.9.5.1.

### Reporting summary

Further information on research design is available in the Nature Portfolio Reporting Summary linked to this article.

### Supplementary information

Supplemental Information

Description of Additional Supplementary Files

Supplementary Data 1

Supplementary Data 2

Supplementary Data 3

Supplementary Data 4

Supplementary Data 5

Supplementary Data 6

Supplementary Data 7

Supplementary Data 8

Supplementary Data 9

Supplementary Data 10

Supplementary Data 11

Supplementary Data 12

Supplementary Data 13

Reporting Summary

## Data Availability

Data that support the findings of this study are available as Supplementary Data files and from the corresponding authors upon reasonable request.
