# Supplementary material for: Molecular hybridization strategy for tuning bioactive peptide function
Source: Commun Biol. 2023 Oct 19;6:1067. doi: 10.1038/s42003-023-05254-7 (PMC10587126; doi:10.1038/s42003-023-05254-7)
Supplement: Supplementary file 1 — Supplemental Information [file 42003_2023_5254_MOESM1_ESM.pdf]

Supporting Information:

Molecular hybridization strategy for tuning bioactive peptide function

Cibele Nicolaski Pedron, Marcelo Der Torossian Torres, Cyntia Silva Oliveira, Adriana Farias Silva, Gislaine Patricia Andrade, Yiming Wang, Maria Aparecida Silva Pinhal, Giselle Cerchiaro, Pedro Ismael da Silva Junior, Fernanda Dias da Silva, Ravi Radhakrishnan, Cesar de la Fuente-Nunez*, Vani Xavier Oliveira Junior*

Table S1. Biological activity and structural characterization of the peptides.

Peptide	MIC (µmol L^{-1})							MHC (µmol L^{-1})	MCF-7 (µmol L^{-1})		*P. gallinaceum* (% sporozoites at 0.8 µmol L^{-1})
	M. luteus **A270**	***S. epidermidis*** **ATCC 12228**	***B. megaterium*** **ATCC 10778**	***S. marcescens*** **ATCC 4112**	***E. cloacae*** **β-12**	***C. albicans*** **MDM8**	***C. tropicalis*** **IOC 4560**		**4 h LD_{50}**	**24 h LD_{50}**	
VmCT1	0.8	3.1	1.6	0.4	>50	12.5	12.5	6.3	50	12.5	93
Anoplin	1.6	6.3	0.8	6.3	3.1	1.6	1.6	50	NT	NT	90
AV	6.3	>50	>50	50	>50	50	50	>100	NT	NT	90
Protonectin	3.1	25	3.1	6.3	25	12.5	12.5	12.5	25	25	26
PV	3.1	>50	12.5	12.5	>50	12.5	6.3	25	-	-	41
VP	>50	>50	>50	>50	>50	>50	>50	>100	-	-	92
Decoralin	0.8	6.3	1.6	3.1	6.3	0.8	1.6	1.6	-	-	0
DV	>50	>50	>50	>50	>50	>50	>50	0.1	-	-	31
VD	25	>50	6.3	12.5	>50	25	6.3	>100	-	-	0
Temporin A	3.1	>50	6.3	12.5	>50	25	12.5	0.2	50	50	15
TV	6.3	>50	6.3	6.3	>50	25	3.1	0.2	-	-	83
VT	3.1	>50	6.3	12.5	>50	12.5	25	0.2	-	-	2

MHC value was considered the concentration that there were approximately 0% of hemolytic activity.

NT: the peptide was not tested.

Table S2. Antimicrobial activity of the peptides against several ESKAPE pathogens.

Peptide	MIC (µmol L^{-1})							
	E. coli **ATCC11775**	*P. aeruginosa* **PAO1**	*P. aeruginosa* **PA14**	*S. aureus* **ATCC12600**	*E. coli* **AIC221**	*E. coli* **AIC222**	*K. pneumoniae* **ATCC13883**	*A. baumannii* **ATCC19606**
VmCT1	16	128	128	8	32	16	64	8
Protonectin	16	128	128	8	16	8	64	16
PV	32	128	128	16	32	16	64	32
VP	128	128	128	128	128	128	128	128
Temporin A	64	128	128	8	128	32	128	32
TV	32	128	128	16	16	8	16	16
VT	64	128	128	4	128	32	128	16

Table S3. Helical fraction of the wild-type and analogs in seven different media calculated by using Lifson-Roig helix-coil theory.[77]

Peptide	Helical fraction (f_H)						
	Water	PBS	SDS	TFE/Water	POPC	POPC:DOPE	POPC:POPG
VmCT1	0.05	0.03	0.17	0.30	0.42	0.55	0.25
Anoplin	0.06	0.10	0.71	0.58	0.05	0.12	0.30
AV	0.08	0.05	0.31	0.42	0.03	0.08	0.04
Protonectin	0.10	0.07	0.69	0.71	0.33	0.58	0.64
PV	0.13	0.08	0.60	0.68	0.44	0.28	0.63
VP	0.01	0	0.12	0.07	0.07	0.02	0.30
Decoralin	0.05	0.06	0.22	0.29	0.23	0.19	0.40
DV	0.11	0.12	0.62	0.90	0.08	0.11	0.41
VD	0.05	0	0.20	0.14	0.10	0.09	0.04
Temporin A	0.08	0.07	0.47	0.65	0.31	0.44	0.19
TV	0.04	0.04	0.28	0.50	0.12	0.35	0.06
VT	0.21	0.18	0.82	1.00	0.45	0.42	1.00

Phosphate buffer saline (PBS, 10 mmol L^{-1}, pH 7.4), sodium dodecyl sulfate (SDS 20 mmol L^{-1}) in water, 2,2,2-trifluoroethanol (TFE) in water (3:2, v:v), palmitoyloleoylphosphatidylcholine (POPC; 10 mmol L^{-1}), palmitoyloleoylphosphatidylcholine: dioleylphosphatidylethanolamine (POPC:DOPE; 3:1, mol:mol, 10 mmol L^{-1}) and palmitoyloleoylphosphatidylcholine: palmitoyloleoylphosphatidylglycerol (POPC:POPG; 3:1, mol:mol, 10 mmol L^{-1}).

Table S4. Resistance to degradation studies. Peptides were exposed to fetal bovine serum enzymes for 6 h and the remaining peptide was calculated based on liquid chromatography coupled to mass spectrometry experiments.

Peptide	30 min	1 h	2 h	4 h	6 h
VmCT1	~ 20%	< 10%	< 10%	-	-
Anoplin	~ 20%	~ 20%	~ 10%	-	-
AV	~ 40%	~ 40%	~ 40%	~ 40%	-
Protonectin	-	-	-	-	-
PV	~ 90%	-	-	-	-
VP	~ 20%	< 20%	< 10%	-	-
Decoralin	~ 70%	< 30%	< 30%	-	-
DV	~ 50%	~ 50%	~ 50%	~ 50%	~ 50%
VD	~ 10%	-	-	-	-
Temporin A	-	-	-	-	-
TV	-	-	-	-	-
VT	-	-	-	-	-

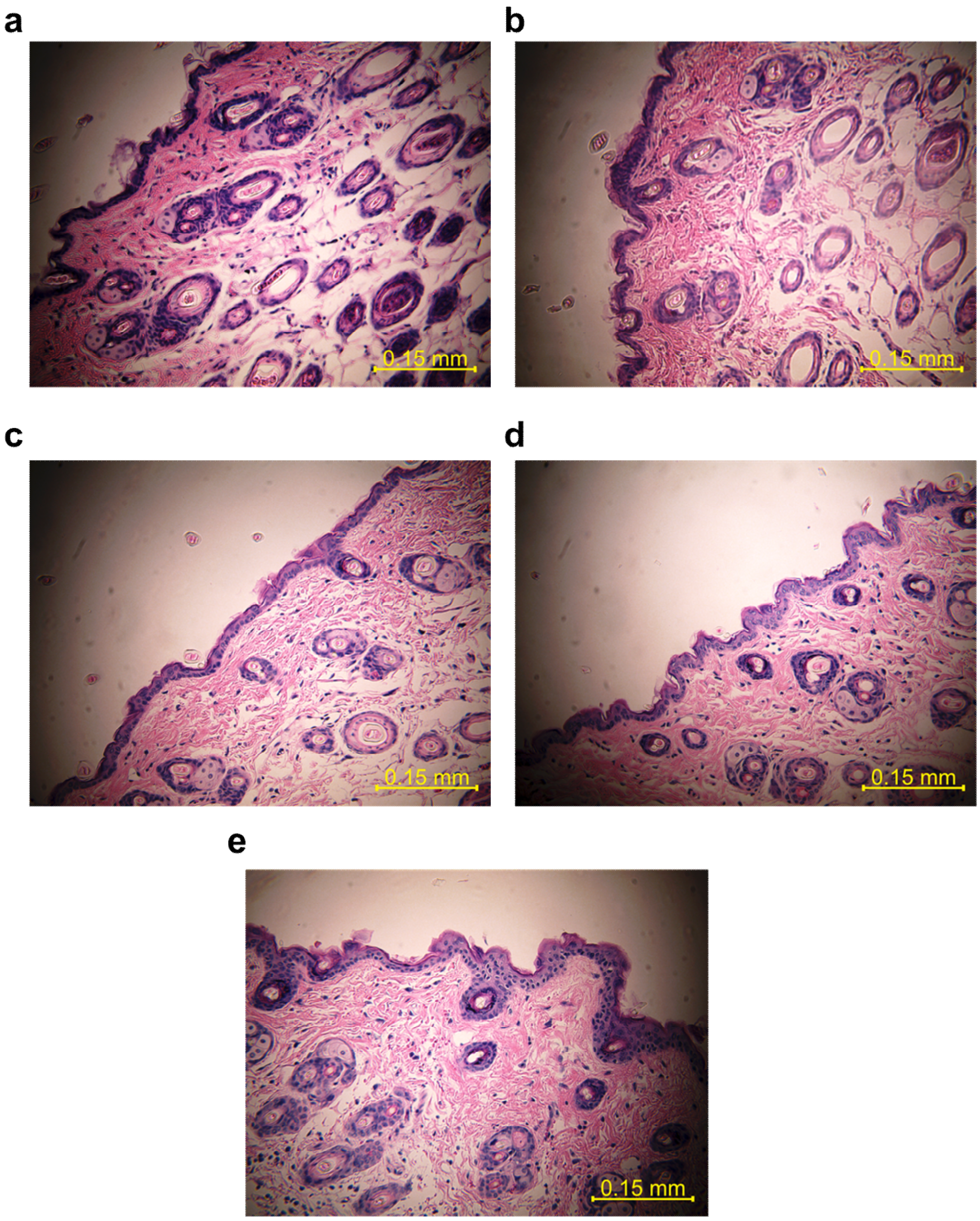

Figure S1. Histopathological analysis of the murine skin tissue after the scarification procedure. The mice were shaved, and a scarification was performed with the tip of a needle on their back. Next, mice were **(a)** infected with *A. baumannii* or **(b)** left untreated. Samples were treated with either **(c)** VmCT1 (8 μmol L^{-1}), **(d)** TV (16 μmol L^{-1}), and **(e)** Polymyxin B (20 μmol L^{-1}). One representative image for each condition is shown in the figure.

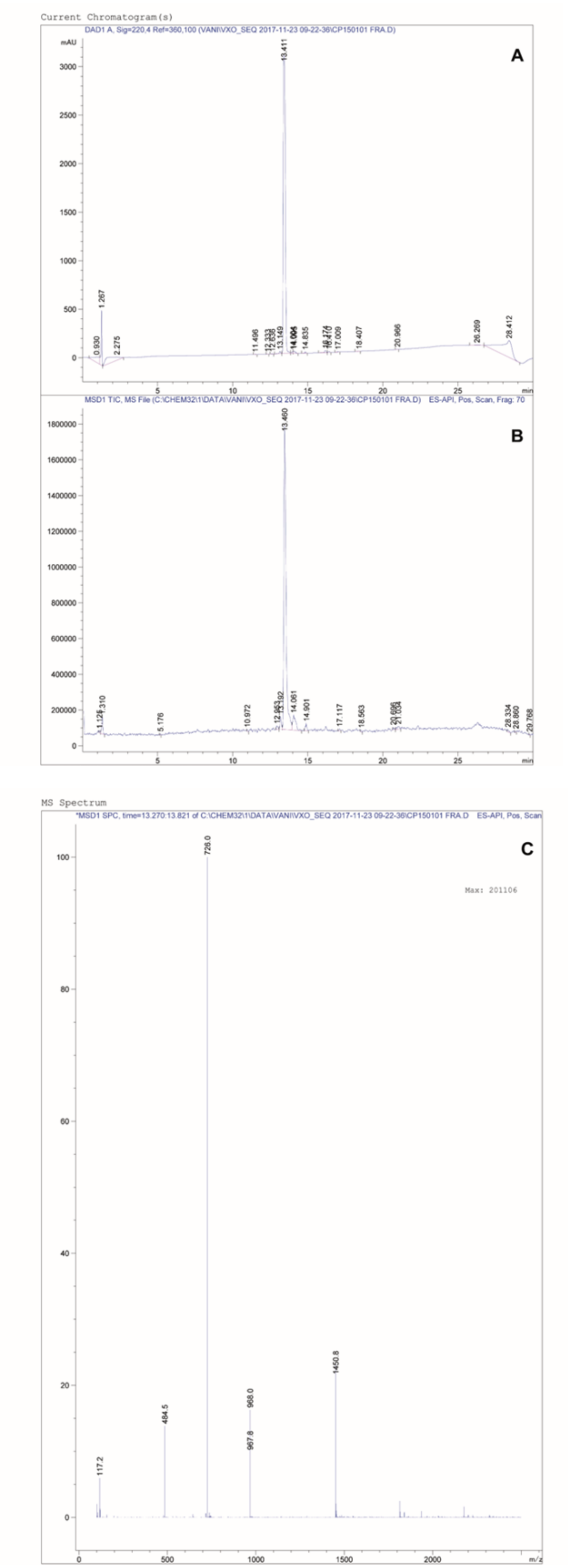

Figure S2. A) Chromatogram, B) Mass spectrum and C) Mass Scan of VmCT1 from 13.270 to 13.821 min at the conditions specified in the Experimental Section.

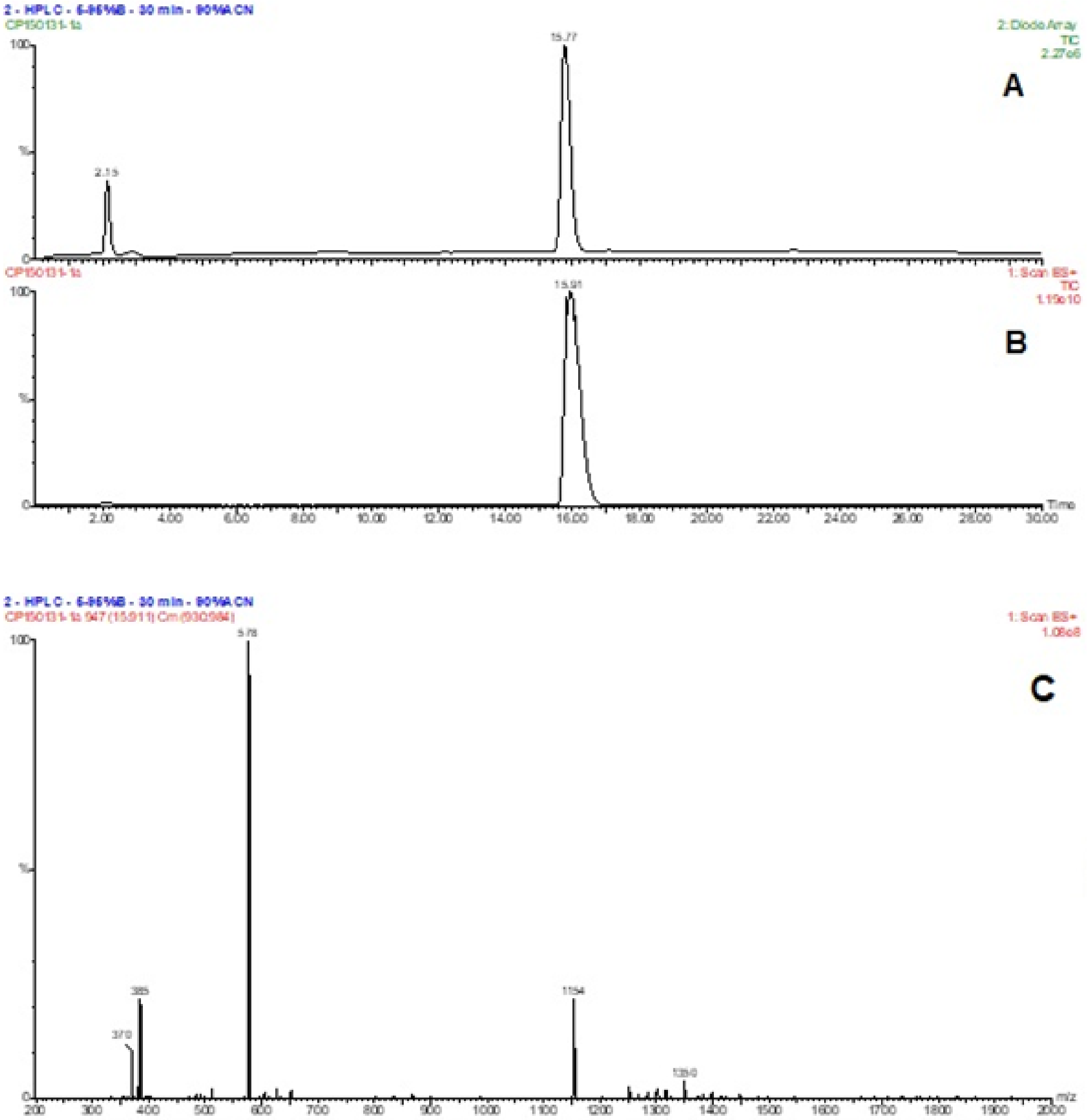

Figure S3. A) Chromatogram, B) Mass spectrum and C) Mass Scan of Anoplin from 15.911 min at the conditions specified in the Experimental Section.

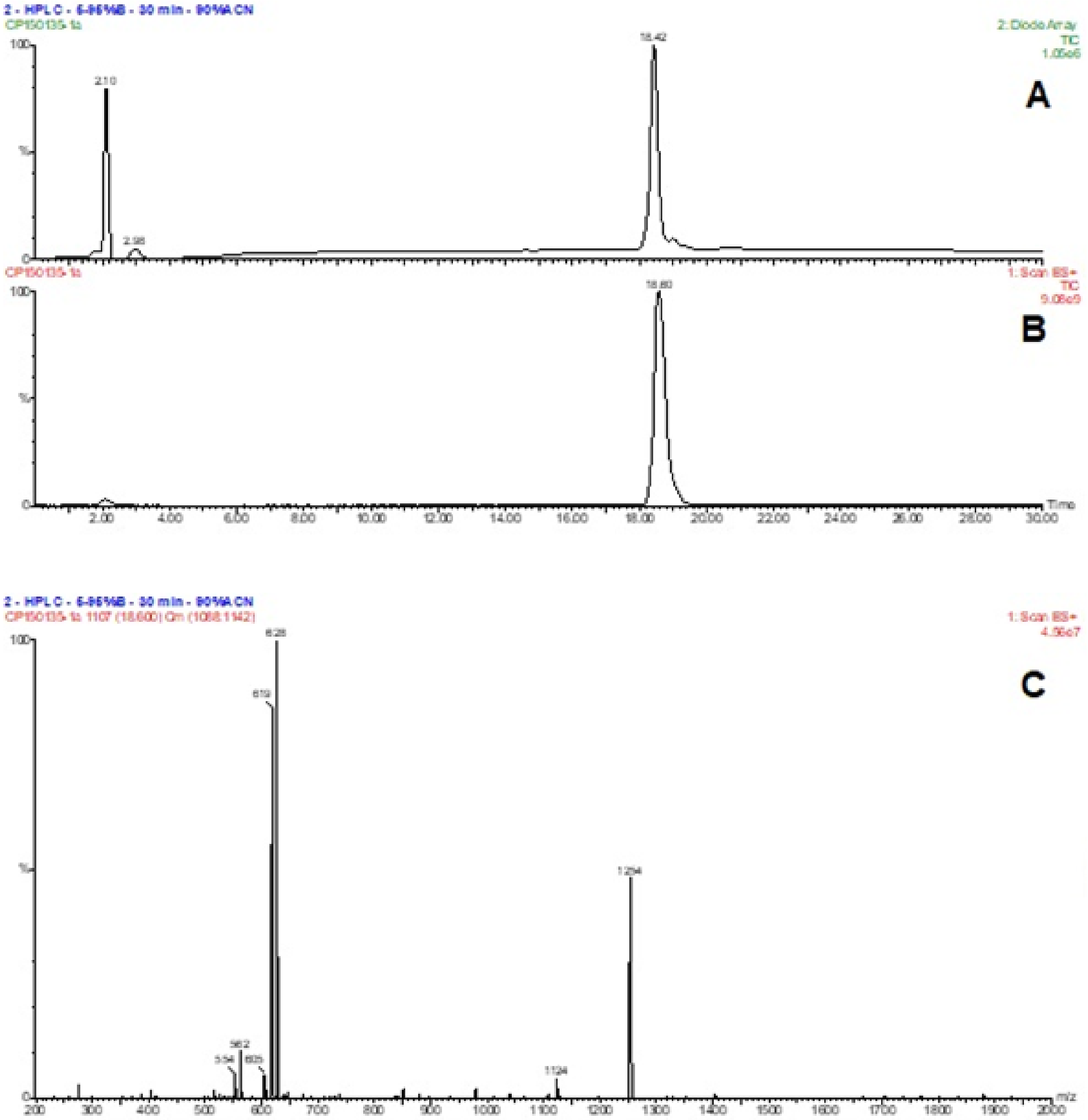

Figure S4. A) Chromatogram, B) Mass spectrum and C) Mass Scan of AV from 18.600 min at the conditions specified in the Experimental Section.

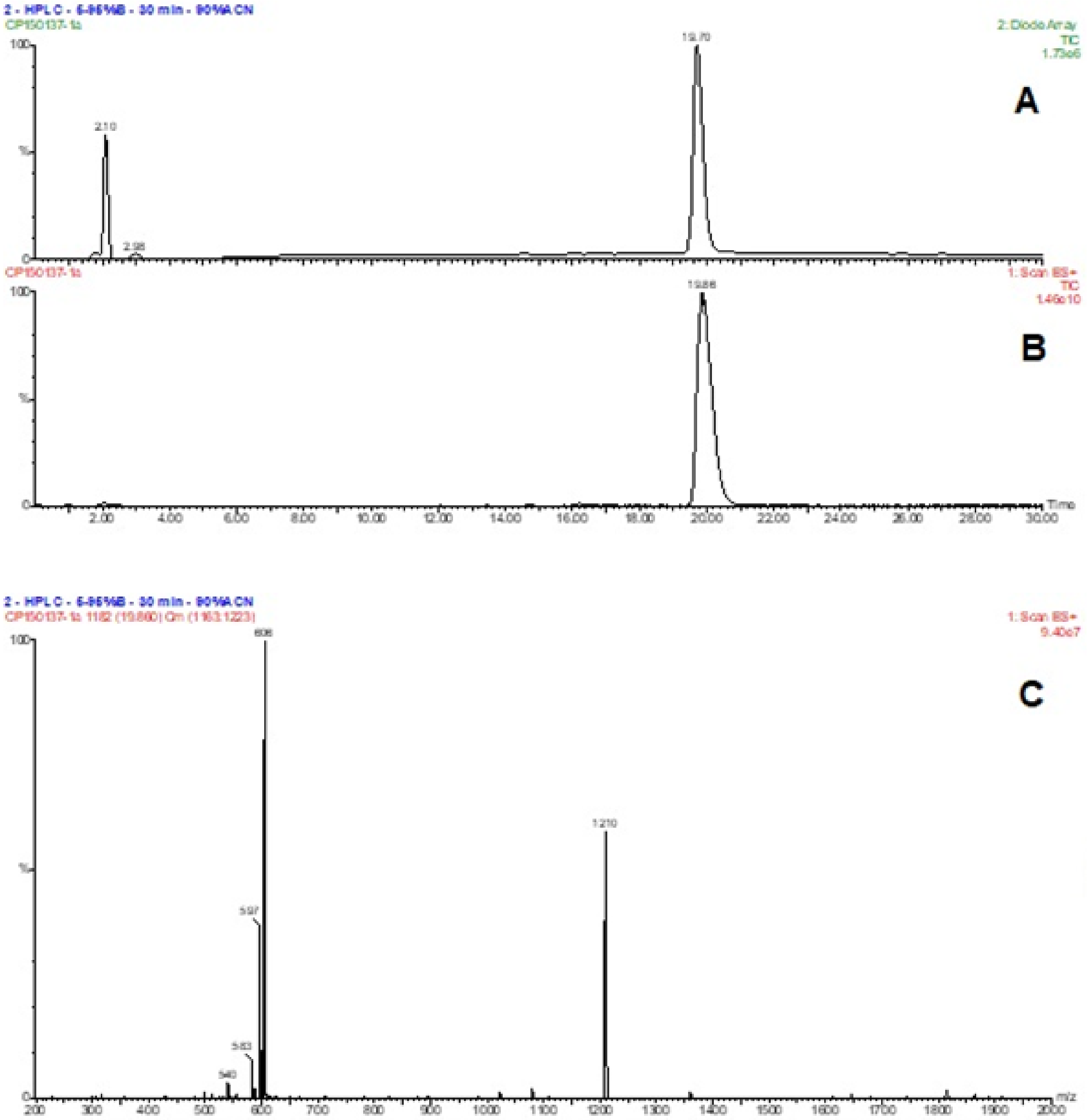

Figure S5. A) Chromatogram, B) Mass spectrum and C) Mass Scan of Protonectin from 19.860 min at the conditions specified in the Experimental Section.

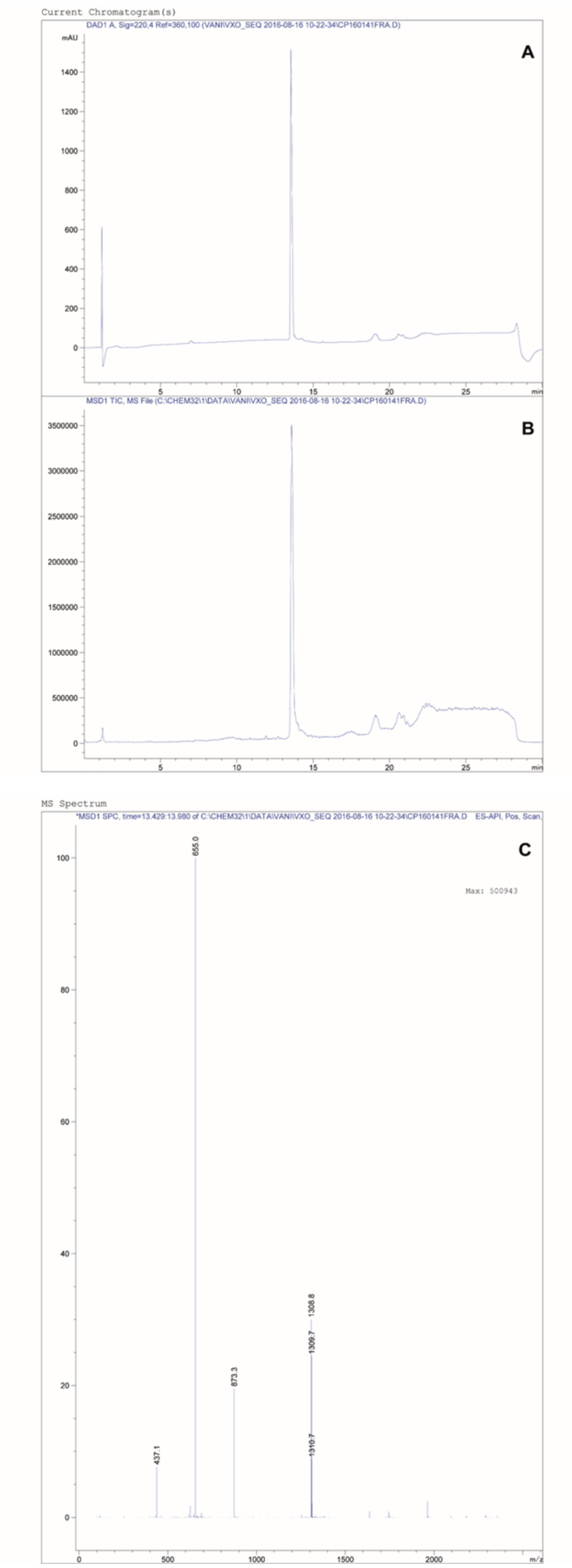

Figure S6. A) Chromatogram, B) Mass spectrum and C) Mass Scan of PV from 13.429 to 13.980 min at the conditions specified in the Experimental Section.

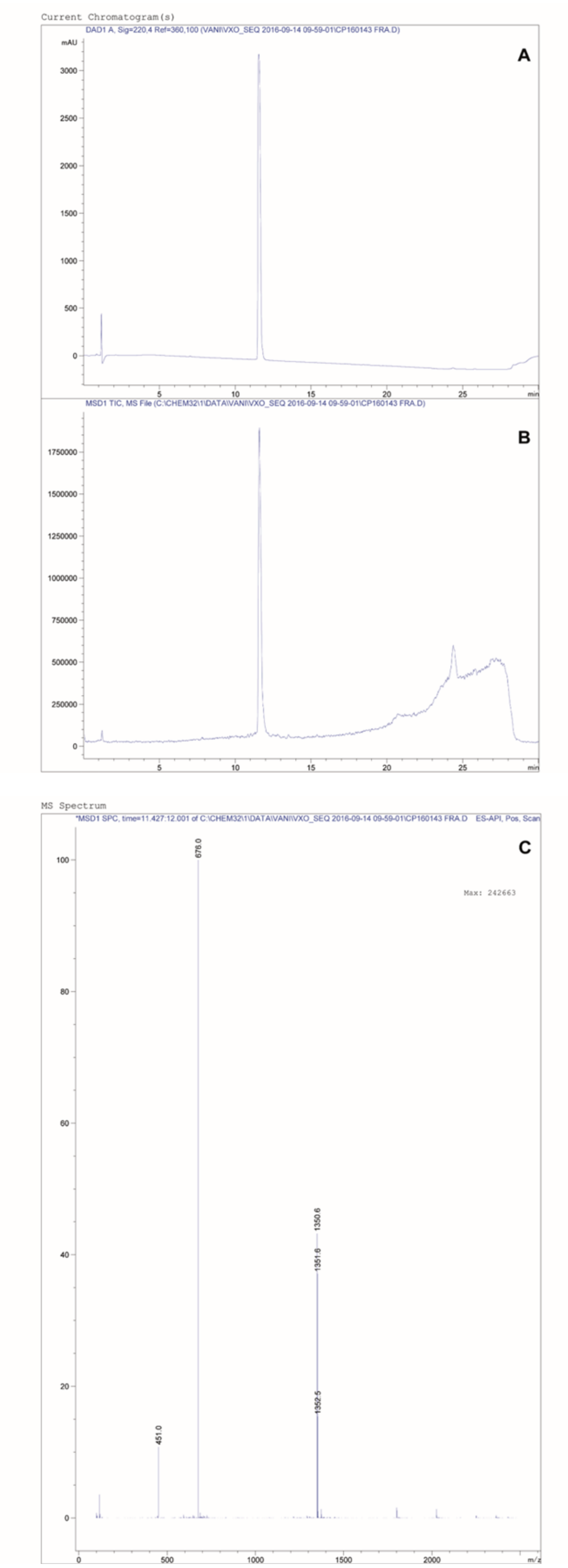

Figure S7. A) Chromatogram, B) Mass spectrum and C) Mass Scan of PV from 11.427 to 12.001 min at the conditions specified in the Experimental Section.

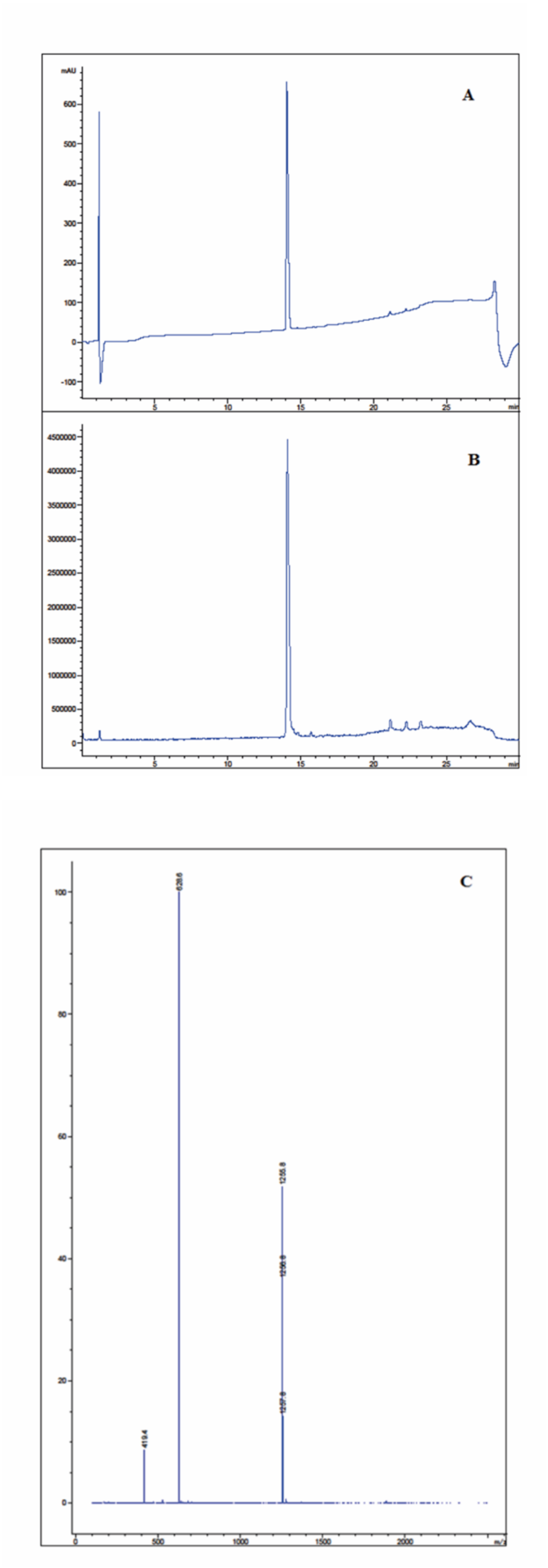

Figure S8. A) Chromatogram, B) Mass spectrum and C) Mass Scan of Decoralin at the conditions specified in the Experimental Section.

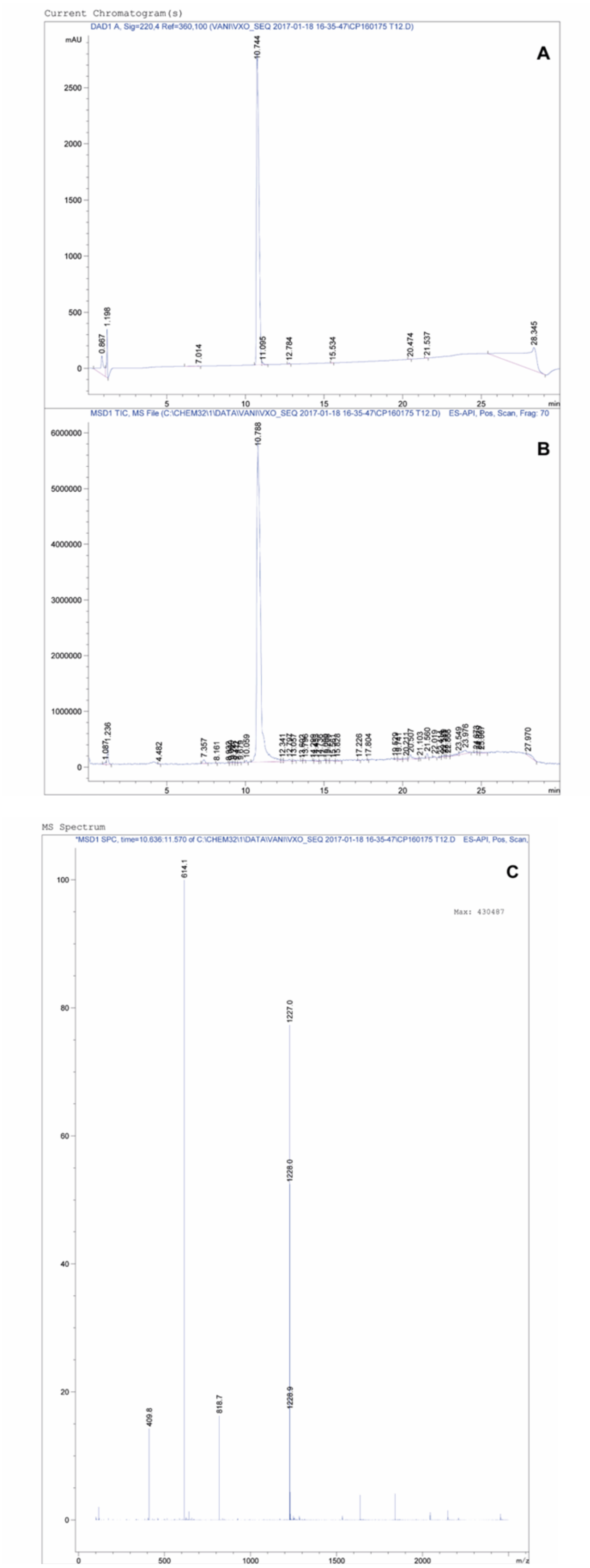

Figure S9. A) Chromatogram, B) Mass spectrum and C) Mass Scan of DV from 10.636 to 11.570 min at the conditions specified in the Experimental Section.

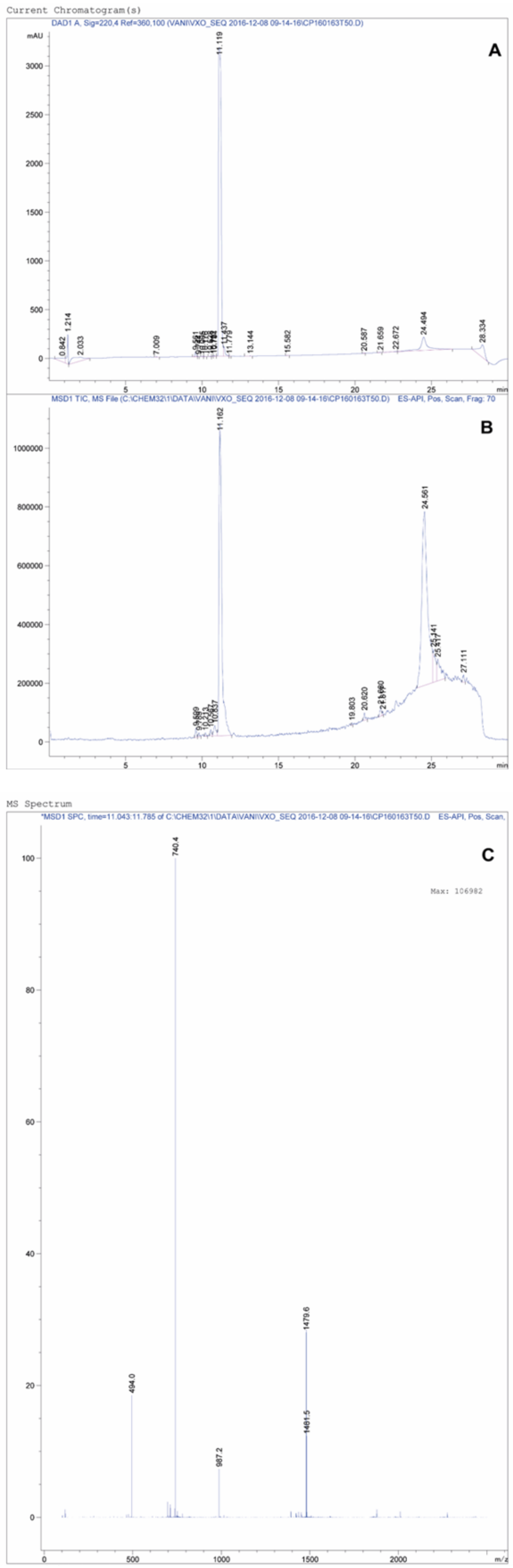

Figure S10. A) Chromatogram, B) Mass spectrum and C) Mass Scan of VD from 11.043 to 11.785 min at the conditions specified in the Experimental Section.

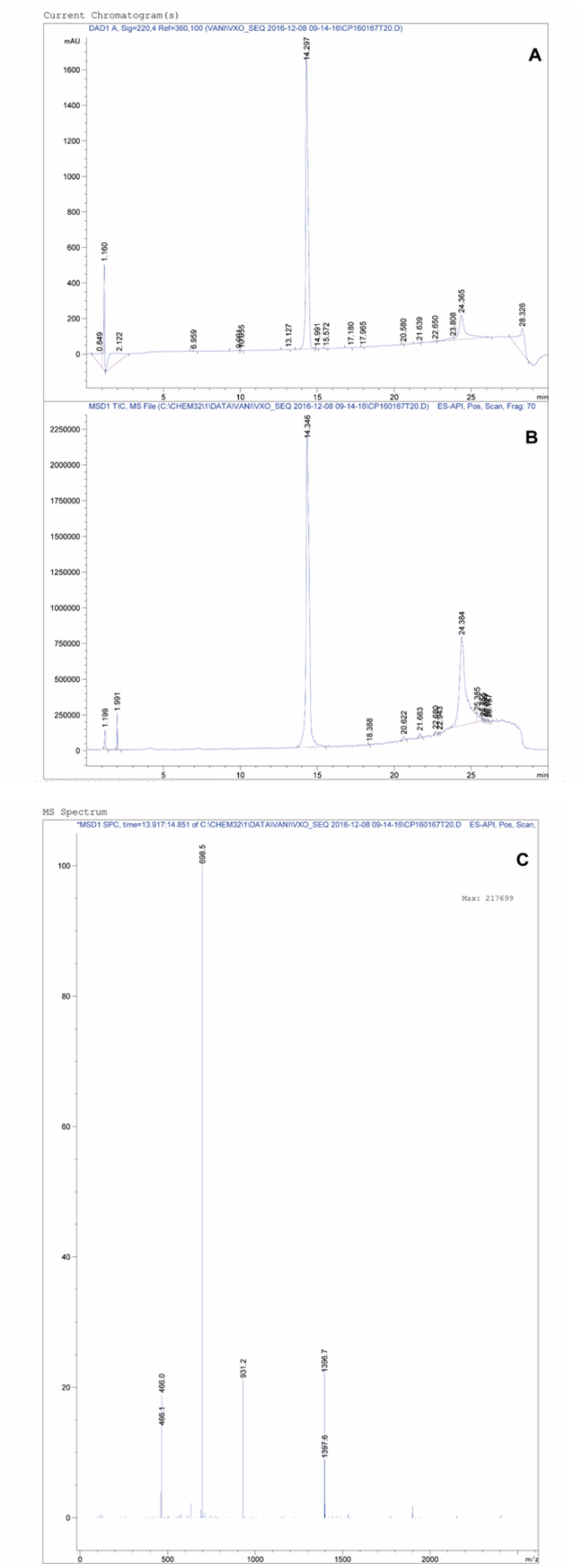

Figure S11. A) Chromatogram, B) Mass spectrum and C) Mass Scan of Temporin A from 13.917 to 14.851 min at the conditions specified in the Experimental Section.

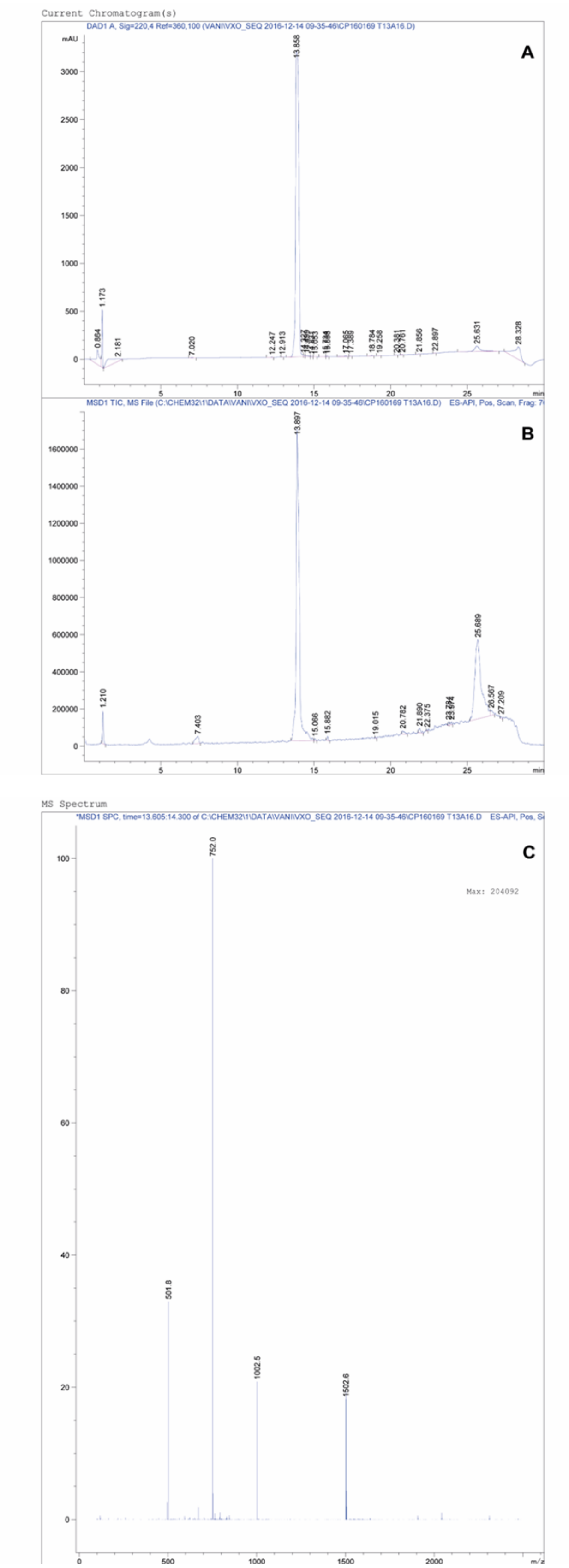

Figure S12. A) Chromatogram, B) Mass spectrum and C) Mass Scan of VT from 13.605 to 14.300 min at the conditions specified in the Experimental Section.

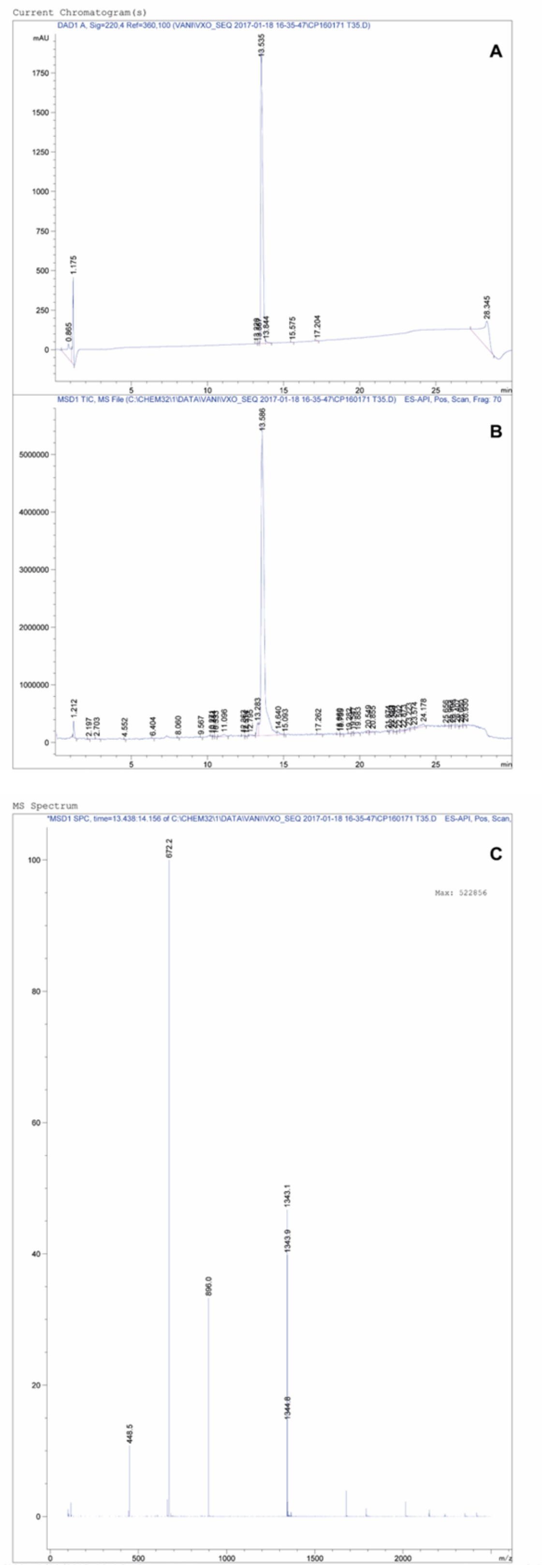

Figure S13. A) Chromatogram, B) Mass spectrum and C) Mass Scan of TV from 13.438 to 14.156 min at the conditions specified in the Experimental Section.
