# Supplementary material for: Molecular hybridization strategy for tuning bioactive peptide function
Source: Commun Biol. 2023 Oct 19;6:1067. doi: 10.1038/s42003-023-05254-7 (PMC10587126; doi:10.1038/s42003-023-05254-7)
Supplement: Supplementary file 2 — Description of Additional Supplementary Files [file 42003_2023_5254_MOESM2_ESM.pdf]

Description of Additional Supplementary Files

File name: Supplementary Data 1
Description: Hemolytic activity of hybrid peptides and templates.

File name: Supplementary Data 2
Description: Circular dichroism spectra of the hybrid peptides and templates in water.

File name: Supplementary Data 3
Description: Circular dichroism spectra of the hybrid peptides and templates in PBS.

File name: Supplementary Data 4
Description: Circular dichroism spectra of the hybrid peptides and templates in SDS.

File name: Supplementary Data 5
Description: Circular dichroism spectra of the hybrid peptides and templates in POPC.

File name: Supplementary Data 6
Description: Circular dichroism spectra of the hybrid peptides and templates in POPC:POPG.

File name: Supplementary Data 7
Description: Circular dichroism spectra of the hybrid peptides and templates in POPC:DOPE.

File name: Supplementary Data 8
Description: Circular dichroism spectra of the hybrid peptides and templates in TFE/water.

File name: Supplementary Data 9
Description: Resistance to proteolytic degradation of hybrid peptides and templates.

File name: Supplementary Data 10
Description: Damage and permeabilization of the bacterial outer membrane by the peptides using the NPN assay.

File name: Supplementary Data 11
Description: Effect of the peptides on the depolarization of the cytoplasmic membrane using the DiSC3(5) assay.

File name: Supplementary Data 12
Description: Anti-infective activity of VmCT1, Temporin A, VT, and TV peptides in a mouse model of A. baumannii skin infection as detailed in the methodology section**.**

File name: Supplementary Data 13
Description: Mouse body weight changes over the experiments.
